# Pandemic Puppies: Demographic Characteristics, Health and Early Life Experiences of Puppies Acquired during the 2020 Phase of the COVID-19 Pandemic in the UK

**DOI:** 10.3390/ani12050629

**Published:** 2022-03-02

**Authors:** Claire L. Brand, Dan G. O’Neill, Zoe Belshaw, Camilla L. Pegram, Kim B. Stevens, Rowena M. A. Packer

**Affiliations:** 1 Department of Clinical Science and Services, The Royal Veterinary College, Hawkshead Lane, North Mymms, Hatfield, Herts AL9 7TA, UK; clbrand@rvc.ac.uk; 2 Department of Pathobiology and Population Sciences, The Royal Veterinary College, Hawkshead Lane, North Mymms, Hatfield, Herts AL9 7TA, UK; doneill@rvc.ac.uk (D.G.O.); cpegram@rvc.ac.uk (C.L.P.); kstevens@rvc.ac.uk (K.B.S.); 3 EviVet Evidence-Based Veterinary Consultancy, Nottingham NG2 5HU, UK; z.belshaw.97@cantab.net

**Keywords:** dogs, puppy, COVID-19, lockdown, welfare, behaviour

## Abstract

**Simple Summary:**

During the early 2020 phase of the COVID-19 pandemic, the UK witnessed an unprecedented increase in puppy acquisition. This led to concerns that these puppies may have missed out on key socialisation experiences during periods of lockdown which could impact their future welfare. This study examined early-life exposure to socialisation and habituation experiences, including social and non-social stimuli and attendance at puppy classes, along with the health and demographics of puppies purchased under 16 weeks of age in the UK between 23 March–31 December 2020 (“Pandemic Puppies” cohort; *n* = 4369). Owners of puppies purchased under 16 weeks of age during the same date range pre-pandemic in 2019 (“2019 puppies” cohort; *n* = 1148) were surveyed as a comparison group. Pandemic Puppies were significantly less likely to have attended puppy training classes or been exposed to visitors to their home before 16 weeks of age than 2019 puppies. Pandemic Puppies were also significantly less likely to have had a veterinary health check prior to purchase or to be Kennel Club registered. In contrast, Pandemic Puppies were more likely to be a ‘Designer Crossbreed’ and to be sold with a passport. This suggests majors shifts in the socialisation and demographics of the puppies acquired during the 2020 phase of the COVID-19 pandemic in the UK and raises concerns for the future welfare of Pandemic Puppies.

**Abstract:**

The UK recorded sharp rises in puppy purchasing during the 2020 phase of the COVID-19 pandemic, with many first-time dog owners purchasing puppies to improve their mental health during this challenging period. Government restrictions on movement and social interaction during the pandemic led to animal welfare concerns over puppies’ reduced time-sensitive exposures to key environmental and social stimuli during their critical developmental period. This study aimed to compare demographics, health and early-life experiences of puppies purchased and brought home < 16 weeks of age between 23 March–31 December 2020 (“Pandemic Puppies”), with dogs purchased and brought home < 16 weeks during the same date period in 2019 (“2019 puppies”). An online survey of UK-based puppy owners was conducted between 10 November and 31 December 2020 with valid responses representing 5517 puppies (Pandemic Puppies: *n* = 4369; 2019 puppies: *n* = 1148). Multivariable logistic regression modelling revealed that Pandemic Puppies were less likely to have attended puppy training classes (67.9% 2019 vs. 28.9% 2020; *p* < 0.001) or had visitors to their home (94.5% 2019 vs. 81.8% 2020; *p* < 0.001) aged < 16 weeks compared with 2019 puppies. Fewer Pandemic Puppies underwent veterinary checks prior to purchase than 2019 puppies (2019: 91.3% vs. 2020: 87.4%; *p* < 0.001), but more were sold with a passport (2019: 4.1% vs. 2020: 7.1%; *p* < 0.001). Pandemic Puppies were significantly more likely to be ‘Designer Crossbreeds’ (2019: 18.8% vs. 2020: 26.1%; *p* < 0.001) and less likely to be Kennel Club registered than 2019 puppies (2019: 58.2% vs. 2020: 46.2%; *p* < 0.001). Greater support from veterinary and animal behavioural professionals is likely needed to ameliorate the health and behavioural impacts of growing up in a pandemic upon this vulnerable population.

## 1. Introduction

The UK recorded sharply rising levels of puppy purchasing during the 2020 phase of the COVID-19 pandemic [1,2,3,4,5], with many people deciding to purchase a puppy for the first time with a view to improving their own and their family’s mental health during this challenging period [1]. Government restrictions to movement during the pandemic (known as “lockdowns”) may have unintentionally promoted sub-optimal puppy purchasing behaviour from UK owners by prohibiting or limiting social contact, and thus thwarting several critical steps in the typical puppy-buying process. A major UK study has previously reported that, during the 2020 phase of the COVID-19 pandemic, greater numbers of puppies were purchased without being viewed in-person in advance of being taken home, or seen within their home environment with their mother and littermates when they were collected, compared to pre-pandemic puppies bought in 2019 [1]. Instead, during the pandemic, puppies were more likely to be viewed virtually (e.g., online video calls or pre-recorded videos/photos), and to be collected from outside their breeder’s property, at a meeting place between the breeder’s and new owner’s properties or delivered directly to their new owner [1]. These changes to the puppy-buying process risked prospective owners purchasing puppies from breeders who may have been using pandemic restrictions as a ‘smokescreen’ to either hide the unsuitable environments that puppies were raised in, or as a cover-up for the illegal importation of puppies from outside the UK to meet demand [6].

In addition to the canine welfare issues related to altered puppy purchasing behaviours, concerns have been raised that the puppies purchased during the 2020 phase of the COVID-19 pandemic may have received inappropriate or insufficient socialisation and habituation during their sensitive period for optimal emotional and behavioural development [7,8,9]. This raises long-term concerns, as this early period is crucial in the development of adult behaviour, including the development of behavioural issues. Recent large cohort studies examining the impact of puppy socialisation on adult behaviour have suggested that limited exposure to a range of experiences between 7–16 weeks can increase the probability of a dog displaying non-social fear such as noise phobias [10] and social fearfulness of other dogs and strangers [11]. Furthermore, poor socialisation increases the risk of aggressive behaviour in dogs [12]. Pandemic Puppies have been speculated to be at an increased risk of developing separation-related behaviours (SRBs), based on assumptions that this cohort of puppies are less likely to have been left alone for significant periods of time during the pandemic due to ‘stay at home’ restrictions, and thus not habituated to being left alone [3]. Prior to the pandemic, SRBs had been reported to affect up to 50% of dogs in the UK [13] but this may even be an underestimate as, by definition, SRBs are demonstrated during the owner’s absence and therefore may not be noticed by the owner. In summary, the early-life experiences of Pandemic Puppies could put this cohort at greater risk of future behavioural issues, which may in turn increase their risk of later relinquishment or euthanasia, given that undesirable behaviours are one of the main drivers for relinquishment [14] and euthanasia of dogs under the age of three years [15,16].

Data on the impact of the COVID-19 pandemic on puppy development are sparse; however, the recent PDSA Animal Wellbeing (PAW) Report 2021 [17] indicated that 27% of dogs acquired since March 2020 displayed behaviours that could be related to a deficit in socialisation and 18% of dogs acquired since March 2020 were displaying signs of distress when left alone. Although several other studies have been published regarding the impact of the COVID-19 pandemic on canine lifestyles, welfare and behaviour in the UK, these studies have largely focused upon those dogs that were already owned pre-pandemic [18,19,20,21]. Thus, despite increasing concern over this vulnerable population of newly purchased Pandemic Puppies, data on the impact of the pandemic on the behaviour and health of Pandemic Puppies purchased during this time are limited.

This study aimed to explore the impact of the 2020 phase of the COVID-19 pandemic on puppy early-life behaviour, socialisation/habituation experiences, and health in the UK. Using a cross-sectional study design, we sought to characterise the demographics, health and behaviour of “Pandemic Puppies” purchased from 23 March–31 December 2020 soon after acquisition, and to assess the actual and intended socialisation and habituation activities of these puppies while aged under 16 weeks. To gain greater inference from these data, we aimed to compare these results with those of puppies purchased during the same date period in 2019 (“2019 puppies”).

## 2. Materials and Methods

### 2.1. Study Design

An online questionnaire including four broad sections was designed iteratively amongst the authors, piloted on a small number of respondents and hosted using the SurveyMonkey platform, as described previously [1]. Briefly, the survey explored (i) puppy demographics, including age at purchase, breed/crossbreed, sex and neuter status; (ii) health, including preventative healthcare provisions such as vaccination and insurance as well as ill-health soon after being brought home; (iii) behaviour both at the time of being brought home and at the point of the survey; and (iv) puppy socialisation experiences from acquisition up to 16 weeks of age.

The pre-purchase motivations, behaviours and purchase behaviours of all owners, as well as the influence of the pandemic on purchasing decisions of Pandemic Puppy owners were also examined and have been reported previously [1].

The inclusion criteria for the survey required participants to be aged over 18 years, resident in the UK and to have purchased a puppy of any breed or crossbreed and brought them home under the age of 16 weeks during 2020 or 2019. Where participants had acquired more than one puppy over the time period, they were asked to answer for the youngest, rather than fill the survey in multiple times. In the case of littermates, they were asked to answer for the dog whose name came first alphabetically. Responses analysed in the current paper are limited to those who purchased their puppy between 23 March–31 December 2020 or the same calendar period in 2019. The full survey is included in Supplementary Materials File S1. The study design was approved by the Social Science Research Ethical Review Board at the Royal Veterinary College (URN: SR2020-0259).

### 2.2. Data Collection

The online questionnaire was open from 10 November to 31 December 2020, disseminated using snowball sampling methods via social media, printed press, radio interviews and key stakeholders in both the commercial sector (companion animal insurance companies, canine registration bodies and puppy selling websites) as well as the charity sector (animal welfare charities and rehoming organisations).

### 2.3. Data Cleaning

The raw survey data were exported from SurveyMonkey into Microsoft Excel to allow for manual data cleaning prior to analysis. This multi-step cleaning procedure involved removing responses from duplicated IP addresses (where the more complete response was retained); removing responses from individuals who did not meet the inclusion criteria and removing responses without data beyond the consent and inclusion criteria.

### 2.4. Qualitative Content Analysis

Qualitative content analysis of free-text options within the multiple-choice questions was performed as previously described [1]. Briefly, three authors familiarised themselves with the data by reading all free-text responses. Two of these authors independently used an inductive approach to develop a coding framework, and based on those two sets of codes, an overall agreed set of codes was created. Finally, these codes were applied to the free-text responses. Where free-text responses were deemed to fit within the scope of existing, deductive fixed-choice responses, data were back-allocated into that category if not already selected. For the purpose of the current study, the free-text responses were analysed for only one question, when respondents selected ‘Other’: “Did you or someone in your household attend any puppy classes with your puppy/dog before they were 16 weeks old?”. The inductive coding framework for this question can be found in Supplementary Materials File S2.

### 2.5. Health Data Coding

Free-text responses to the health-related question “Soon after you brought your puppy home, did you notice any of the following?” were analysed by C.L.B to extract pertinent disorder information. During this analysis C.L.B. was blinded to whether the responses pertained to 2019 puppies or 2020 puppies. ‘Layperson’ responses were assigned for each separate health issue, as described by the respondent. For example, the text “Very flaky skin. She had dandruff and smelt bad. She was a lot better as soon as we gave her a bath.” was categorised as ‘very flaky skin’ and ‘dandruff’. All categorised layperson terms from the study were then analysed by two authors, veterinary surgeons C.L.P and D.G.O’N, who were also blinded to which acquisition years the layperson terms related to, as part of an objective and established process for VetCompass™ coding of clinical notes [22]. The coding framework used for assigning the free-text responses to the VetCompass™ disorder terms can be found in Supplementary Materials File S3. Briefly, each extracted layperson term was mapped to an appropriate VetCompass™ disorder term (e.g., ‘vomiting’ would map to ‘enteropathy’).

Owners were also presented with a list of pre-defined of health problems, which were also categorised and assigned to VetCompass™ disorder terms [22], with the options of ‘Runny faeces and/or diarrhoea’ and ‘Being sick (vomiting)’ allocated to ‘enteropathy’; the options of ‘Fleas/other parasites visible in fur/skin’ and ‘Worms in faeces’ allocated to ‘parasite infestation’; the options of ‘Hair loss’, ‘Wounds/sore areas of skin’ and ‘Frequent itching/licking’ allocated to ‘skin (cutaneous) disorder’; the option of ‘Runny eye(s)’ allocated to ‘ophthalmological disorder finding’ and finally the option for ‘Coughing’ allocated to ‘upper respiratory tract finding’.

### 2.6. Breed/Crossbreed Categorisation

The breed/crossbreed allocation system used by VetCompass™ [23] was used to construct a pre-populated drop-down menu of over 200 breeds/crossbreeds commonly encountered in the UK. Survey participants could choose the breed/crossbreed term for their dog from this list, or fill in a free-text ‘Not on the list – please specify’ option. For the purposes of this study, purebred dogs were categorised as those having ancestry over many generations of the same breed, and which are recognised as such by The Kennel Club (UK) [24] and/or other international kennel clubs [25,26]. Owners of purebred dogs were asked to report whether their puppy was registered with The Kennel Club, the major UK registration body for pedigree dogs. For puppies to be Kennel Club registered, both of their parents must be registered members of the same breed. This variable was recorded to allow investigation of demographic changes in the UK’s registered and unregistered dog populations during the COVID-19 pandemic, as there is no overall register of UK dogs. ‘Designer’ crossbred dogs were defined as intentional crosses of different breeds/crossbreeds which were reported by the owner using a breed-indicative name; commonly a portmanteau of the parental breeds (e.g., Cockapoo, for a Cocker Spaniel crossed with a Poodle breed). Crossbred dogs were defined as mixed breeds of unknown origin, or crosses who were not reported using a breed-indicative designer crossbreed name, e.g., Spaniel Cross.

### 2.7. Calculation of Typical Adult Bodyweight

A typical adult bodyweight (kg) was assigned to each dog using VetCompass™ data on mean adult bodyweights for each breed or crossbreed/sex combination from data collected on ≥100 dogs as described previously [27]. Body weights were categorised as ≤10.0 kg, 10.0 to <20 kg, 20 to <30 kg, 30 to <40 kg and ≥40 kg. This variable was included in analyses predicting owner behaviour (e.g., attendance of puppy classes) rather than modelling biological effects.

### 2.8. Statistical Analysis

The cleaned data from Excel were imported into IBM SPSS Statistics v27 (SPSS Inc., Chicago, IL, USA). Descriptive statistics (frequency and percentage) were calculated for all categorical variables, with univariable analysis (chi-square; *X^2^*) performed to compare the 2019 puppies and Pandemic Puppies. The only continuous variable examined, of puppy age at survey, was calculated as the mean (standard deviation; SD) and was normally distributed. A multivariable binary logistic regression model was built with online or in-person attendance at puppy classes, (yes vs. no) as the binary outcome. The covariables assessed as risk factors were chosen using an ‘information theory’ approach [28], and included acquisition year, sex, breed/crossbreed categorisation, typical adult bodyweight, neuter status, owner age, owner gender, presence of children in the house, owner’s regional location within the UK and owner’s previous experience with dog ownership. Multicollinearity was assessed by iteratively running collinearity diagnostics, inspecting standard errors for inflation, and examining changes in coefficients when adding new independent variables. Where mild collinearity was detected, independent variables were retained if the model remained stable, as per the information theory approach to model building. The Hosmer–Lemeshow Test was used to evaluate the quality of the model fit. Statistical significance was set at the 5% level.

### 2.9. Spatial Analysis

Respondents were given the option of providing the first half of their postcode to assess how representative of the UK population the study sample was and explore regional trends in puppy demographics. Partial postcodes were checked for validity using the Office for National Statistics (ONS) National Statistics Postcode Lookup (NSPL) May 2021 data [29] and then assigned to one of 12 UK regions [30].

A choropleth map was produced using ArcGIS 10.2 (Environmental Systems Research Institute, Redlands, CA, USA) to visualise regional differences.

## 3. Results

The final analysis reported in this paper included *n* = 5517 puppies (2019: 20.8%, *n* = 1148; 2020: 79.2%, *n* = 4369) following data cleaning as described previously [1].

### 3.1. Owner Demographics

Owner and household demographics have been described elsewhere [1] but briefly, the majority of the respondents to the survey for both 2019 puppies and Pandemic Puppies were female (2019: 92.0% vs. 2020: 90.0%; *X*^2^ = 6.61, *p* = 0.202) and between 25–54 years of age (2019: 48.6% vs. 48.7%; *X*^2^ = 11.77, *p* = 0.067). Pandemic Puppies were significantly more likely to be owned by a first-time dog owner (2019: 33.3% vs. 2020: 40.4%; *X*^2^ = 16.90, *p* < 0.001).

### 3.2. Puppy Demographics

Using the date of birth provided by the owner, the mean age of puppies at the time of the survey for each cohort was 514 days (SD ± 92.90) for 2019 puppies (*n* = 1148) and 157 days (SD ± 79.77) for Pandemic Puppies (*n* = 4360). A small number of Pandemic Puppies (2.7%, *n* = 118) were reported by the owners to still be aged under 8 weeks at the time of survey completion. In terms of age at acquisition, 67.3% of Pandemic Puppy owners were told by their puppies’ breeder that their puppy was aged 7–8 weeks at time of sale (out of a possible range of < 6–16 weeks), compared to 52.5% of 2019 puppy owners (*X*^2^ = 113.01, *p* < 0.001). There was no significant difference between the sex distribution of 2019 puppies and Pandemic Puppies (male, 2019: 51.7% vs. 2020, male: 53.4%; *X*^2^ = 1.12, df = 1, *p* = 0.290).

#### 3.2.1. Breed/Crossbreed Characteristics

All free-text responses were allocatable to existing VetCompass™ breeds/crossbreed terms. The 10 most popular dog breeds/crossbreeds among the puppies purchased for each year are described in Table 1. The proportion of designer crossbred dogs increased significantly from 2019 to 2020 (2019: 18.8% vs. 2020: 26.1%; *X*^2^ = 27.67, df = 1, *p* < 0.001) with a corresponding significant decrease in purebred puppies (2019: 78.7% vs. 2020: 70.3%; *X*^2^ = 32.48, df = 1, *p* < 0.001). There was a significant decrease in the proportion of puppies registered with The Kennel Club in the Pandemic Puppy cohort compared to the 2019 puppy cohort (2019: 58.2% vs. 2020: 46.2%; *X*^2^ = 46.20, df = 1, *p* < 0.001).

#### 3.2.2. Spatial Analysis of Designer Crossbreeds in the Pandemic Puppy Cohort

As described elsewhere [1], spatial analysis of respondent postcode data indicated that both the 2019 puppy owner and Pandemic Puppy owner cohorts were highly representative of the UK population distribution when compared to ONS population data for mid-2020.

**Figure 1:**
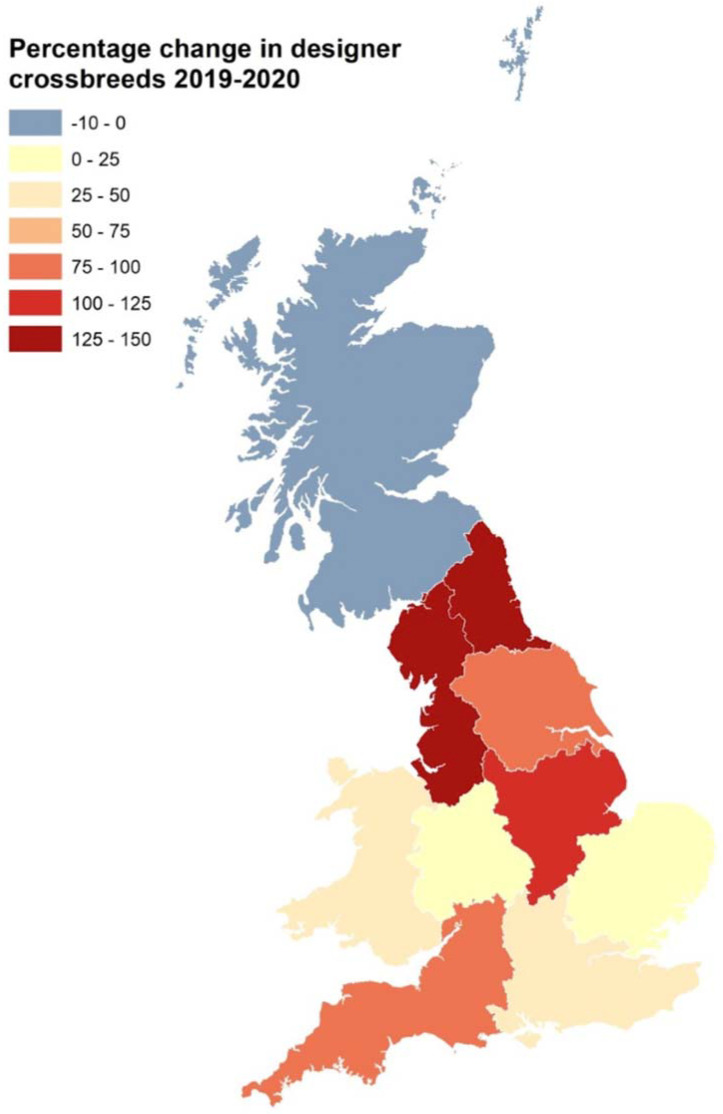
Spatial analysis of the percentage change in the distribution of designer crossbreeds in the Pandemic Puppy cohort (*n* = 3502) relative to the 2019 puppy cohort (*n* = 944). The overall sample size of designer crossbreeds in both 2019 and 2020 in Northern Ireland was too small to draw any meaningful conclusions (2019: *n* = 5; 2020: *n* = 7) and was therefore omitted from this analysis.

The popularity of designer crossbred dogs in the Pandemic Puppy cohort compared to the 2019 puppy cohort was further explored via spatial analysis. Overall, in both the 2019 puppy cohort and the Pandemic Puppy cohort, designer crossbreeds were most popular in London (2019: 25.4% and 2020: 33.3%). Figure 1 indicates an increase in popularity in designer crossbred puppies relative to purebred and crossbred puppies during 2020 in the North West and North East (+129.7% and +133.0%, respectively) when compared to the 2019 puppy cohort.

### 3.3. Breeder Provisions

Pandemic Puppies were significantly less likely to have had a health check by a veterinary surgeon prior to being taken home by their owners than 2019 puppies (2019: 91.3 & vs. 2020: 87.4%; Table 2). In addition, Pandemic Puppies were significantly less likely to have received their second vaccinations prior to being taken home compared to 2019 puppies (2019: 8.4% vs. 2020: 6.4%; Table 2). Pandemic Puppies were significantly more likely to have a Pet Passport at the time they were taken home (2019: 4.1% vs. 2020: 7.1%; Table 2), and their owners were significantly less likely to receive a copy of their puppy’s pedigree (2019: 61.7% vs. 2020: 47.3%; Table 2) or Kennel Club change of ownership form than 2019 puppy owners (2019: 59.4% vs. 2020: 43.6%; Table 2). Breeders of Pandemic Puppies were more likely to have offered owners advice on diet (2019: 61.6% vs. 2020: 76.9%; Table 2), health (2019: 49.2% vs. 2020: 58.0%; Table 2), exercise (2019: 32.5% vs. 2020: 37.8%; Table 2) and training/behaviour (2019: 42.1% vs. 2020: 50.8%; Table 2) compared to breeders’ of 2019 puppies. There was no significant difference between the proportion of puppies microchipped prior to being taken home between the two years, although only 92.4% of 2019 puppies and 93.2% of Pandemic Puppies had had this legal requirement fulfilled (Table 2).

The relationship between being sold with a Pet Passport and age when taken home were explored in both cohorts (Table 3), in light of legal restrictions on import age in the UK (minimum age 15 weeks at import under both the Pet Travel Regulations [31] and the Balai Directive [32]). Pandemic Puppies were significantly more likely than 2019 puppies to have been sold with a Pet Passport at a younger age (e.g., under the age of 13 weeks; 2019: 72.2% vs. 2020: 87.4%; *X*^2^ = 6.90, *p* = 0.009).

### 3.4. Puppy Health Soon after Acquisition

The majority of puppies in both years were registered with a veterinary surgeon at the time of the survey (2019: 99.5%, *n* = 1072 vs. 2020: 98.5%, *n* = 4098) with no significant difference between the two populations (*X*^2^ = 10.25, *p* = 0.006).

In terms of the health disorders reported soon after the puppies were brought home, the prevalence did not differ between the two cohorts for the individual VetCompass™ disorder terms seen, with the exception of skin disorders and parasite infestation which were both significantly higher in Pandemic Puppies (Table 4). Soon after being brought home, disorder prevalence in the Pandemic Puppy cohort was higher compared to the 2019 puppy cohort, with 18.4% of Pandemic Puppy owners reporting their puppy had one or more individual disorders compared to 15.8% of 2019 puppy owners (*X*^2^ = 4.38, *p* = 0.036).

Insurance levels at the time of the survey did not significantly differ between the 2019 puppies and Pandemic Puppies (83.5% vs. 83.9%, respectively); however, future intentions for insurance differed, with significantly more Pandemic Puppies owners who had not currently, but planned to, insure their puppy in the future compared to 2019 puppy owners, and significantly fewer Pandemic Puppy owners who did not plan to insure their puppy in the future compared to 2019 puppy owners (*X*^2^ = 41.95, df = 4, *p* < 0.001) (Table 5).

The majority of 2019 puppies and Pandemic Puppies had received both first and second vaccinations at the time of the survey (92.7% vs. 82.5%, respectively); however, significantly more Pandemic Puppies had only received their first vaccinations compared with 2019 puppies (*X*^2^ = 72.17, *p* < 0.001; Table 6). The number of owners who indicated that they never plan to vaccinate their puppy did not differ between the two cohorts (2019 puppies: 0.3% vs. Pandemic Puppies: 0.2%).

At the time of the survey, more 2019 puppies had been neutered (43.0%) than Pandemic Puppies (19.5%); however, significantly more Pandemic Puppy owners were planning to have their dogs neutered in the future (2019 puppies: 21.4% vs. Pandemic Puppies: 45.4%) or had not yet decided whether to neuter their dog (2019 puppies: 14.2% vs. Pandemic Puppies: 21.7%) than 2019 puppy owners (*X*^2^ = 383.73, *p* < 0.001; see Table 7).

### 3.5. Puppy Socialisation Experiences and Behaviour Soon after Acquisition

The socialisation experiences of Pandemic Puppies under the age of 16 weeks statistically differed to those of the 2019 puppies in several ways.

#### 3.5.1. Being Left Alone

Pandemic Puppies were significantly less likely than 2019 puppies to have been deliberately left alone for any period of time while aged under 16 weeks (2019 puppies: 81.0% vs. Pandemic Puppies: 74.6%; *X*^2^ = 745.07, *p* < 0.001). However, much of this difference can be explained by some of the 2020 cohort having not yet reached 16 weeks of age at the time of the survey, but their owners intending to leave them alone before this age was reached (9.0%) (Table 8).

At the time of the survey, Pandemic Puppies were significantly less likely than 2019 puppies/dogs to be left alone for more than four hours without being taken for exercise or having someone come in to check on them (2020: 3.1%, vs. 2019: 9.4%; *X*^2^ = 74.77, *p* < 0.001). When Pandemic Puppy owners were asked whether they would be likely to leave their puppy/dog alone for over four hours in the future, the figure increased to 9.8%; however, these levels were still significantly lower when compared with 2019 puppy/dog owners, of which 17.2% felt they were likely to leave their dog alone for over four hours (without being taken for exercise or having someone come in to check on them) in the future (*X*^2^ = 46.12, *p* < 0.001).

#### 3.5.2. Socialisation Experiences under 16 Weeks of Age

Pandemic puppies and 2019 puppies differed in their socialisation experiences aged under 16 weeks in several key areas. Almost three times the number of Pandemic Puppies had not experienced a visitor in their home before the age of 16 weeks (12.8%), compared to 2019 puppies at the same age (4.6%) (Table 9). Conversely, Pandemic Puppies were more likely to have visited, or their owners were planning for them to visit, a groomer aged under 16 weeks than 2019 puppies (2019: 19.9% vs. 2020: 28.7%; Table 9), and were more likely to have experienced fireworks aged under 16 weeks than 2019 puppies (2019: 31.7% vs. 2020: 44.4%; Table 9).

Pandemic Puppies were less likely to have met people or dogs from outside the household, or have walked in a public space and near traffic under the age of 16 weeks than 2019 puppies (Table 9). However, many of these differences could be explained by the young age of some of the Pandemic Puppy cohort, and whose owners intended to expose their dog to these experiences before they reached 16 weeks of age.

#### 3.5.3. Attendance at Puppy Training Classes

Pandemic Puppies were significantly less likely to have attended in-person puppy classes under 16 weeks of age compared with 2019 puppies, with 28.9% having attended puppy classes at the time of the survey, in contrast to 67.9% of 2019 puppies (*X*^2^ = 745.07, *p* < 0.001; Table 10). This difference (39.0%) could not be entirely explained by future intentions to attend puppy classes while puppies were aged under 16 weeks in the Pandemic Puppy cohort (16.6%). Although significantly more puppies had attended online puppy classes in 2020 (2019: 1.0% vs. 2020: 6.7%), in total, 47.8% of Pandemic Puppies did not attend any formal puppy training under the age of 16 weeks, compared to 31.2% of 2019 puppies.

#### 3.5.4. Attendance at Puppy Training Classes: Multivariable Analysis

Multivariable logistic regression modelling identified seven variables significantly associated with attendance at either in-person or online puppy training classes whilst puppies were aged under 16 weeks (Table 11). The Hosmer–Lemeshow Test indicated acceptable model fit (*p* = 0.567).

Pandemic Puppies had a significantly lower odds of attending puppy classes than 2019 puppies (odds ratio (OR) 0.23, 95% CI 0.20 to 0.27, *p* < 0.001). In addition, dogs with a typical adult weight ≤ 10 kg had significantly lower odds of attending puppy classes compared with dogs with a typical adult weight of 10 to < 20 kg (OR 0.65, 95% CI 0.55 to 0.76, *p* < 0.001).

Owner demographics significantly impacted the likelihood of attending puppy classes while their puppy was under 16 weeks. Owners in the youngest age bracket of 18 to 24 years of age had significantly lower odds of taking their puppies to puppy classes compared to owners aged 25 to 34 years of age (OR 0.61, 95% CI 0.46 to 0.81, *p* < 0.001). In contrast, owners aged 35 to 44 and 45 to 54 had significantly increased odds of taking their puppies to puppy classes (OR 1.23, 95% CI 1.01 to 1.50, *p* = 0.037; OR 1.21, 95% CI 1.01 to 1.46, *p* = 0.045, respectively) compared to owners aged 25 to 34 years of age. Male owners had lower odds of taking their puppies to puppy classes compared to female owners (OR 0.75, 95% CI 0.60 to 0.92, *p* = 0.007). Owners with children in the household had lower odds of taking their puppies to puppy classes compared to those with no children in the household (OR 0.86, 95% CI 0.74 to 0.99, *p* = 0.042). Owners with at least one adult in the household having previous dog ownership experience had lower odds of taking their puppies to puppy classes compared to owners in households where all members were first-time dog owners (OR 0.59, 95% CI 0.51 to 0.68, *p* < 0.001). Finally, owners living in the East of England (OR 2.12, 95% CI 1.63 to 2.75, *p* < 0.001), London (OR 1.59, 95% CI 1.19 to 2.11, *p* = 0.001), the South East (OR 1.74, 95% CI 1.35 to 2.25, *p* < 0.001) and the South West (OR 1.67, 95% CI 1.26 to 2.20, *p* < 0.001) had increased odds of taking their puppies to puppy classes compared to owners living in Scotland. In contrast, owners living in Northern Ireland had decreased odds of taking their puppies to puppy classes compared to owners living in Scotland (OR 0.32, 95% CI 0.13 to 0.77, *p* = 0.011).

#### 3.5.5. Puppy Behaviour

At the time of the survey, the most common undesirable behaviours that Pandemic Puppy owners reported were ‘Jumping up at people’ (36.1%), ‘Mouthing’ (33.1%) and ‘Pulling on their lead’ (31.0%). Similarly, the most common undesirable behaviours that 2019 puppy owners reported were ‘Pulling on their lead’ (38.6%), ‘Jumping up at people’ (31.0%), and ‘Chasing e.g., cats, wildlife’ (24.9%). The majority of undesirable behaviours reported by the owners at the time of the survey differed in prevalence between the two cohorts (Table 12), however, given the different developmental stages of the puppies/dogs, this is likely confounded by age and thus statistical comparisons were not performed.

### 3.6. Puppy Relinquishment

At the time of the survey, the vast majority of 2019 puppies and Pandemic Puppies were still owned by their original owner, who had not considered rehoming them. There was no significant difference in the levels of considered or actual relinquishment between 2019 puppies and Pandemic Puppies’ owners (*X*^2^ = 3.79, *p* < 0.580; Table 13).

## 4. Discussion

This study has for the first time described the demographics, health, socialisation and early-life behaviours of ‘Pandemic Puppies’ bought during the 2020 phase of the COVID-19 Pandemic, and has compared these characteristics with 2019 puppies, raised and sold prior to the pandemic. Perhaps unsurprisingly, given the dramatic lifestyle changes that humans experienced during the 2020 phase of the COVID-19 pandemic [33], including prolonged periods of social isolation, the early-life experiences of puppies during that period differed in several key ways compared with ‘pre-pandemic’ 2019 puppies. In particular, Pandemic Puppies were significantly less likely to have experienced visitors to the household, or attended any formal puppy classes under the age of 16 weeks, compared to 2019 puppies.

Demographic differences between 2019 puppies and Pandemic Puppies were also identified, particularly related to breed popularity and the rise of ‘designer crossbreeds’ during the pandemic, which may reflect the differing demographics, motivations for dog ownership and dog-ownership experiences of owners who bought puppies during the 2020 phase of the COVID-19 pandemic, as previously characterised [1].

### 4.1. Socialisation and Habituation Practices

#### 4.1.1. Puppy Classes

Early life exposure to social and non-social stimuli in a calm and controlled manner, while puppies are within their sensitive developmental period is a key practice in preparing puppies for their future life, and reducing the development of behavioural issues [34]. One common practice towards achieving this is attendance of puppy classes, a popular socialisation activity internationally [35,36,37]. Almost half of all Pandemic Puppies did not attend any puppy training classes compared to just under a third of 2019 puppies. After accounting for confounding factors, Pandemic Puppies had significantly lower odds of attending puppy classes compared with 2019 puppies. This difference is likely to have been influenced by face-to-face social contact outside of household ‘bubbles’ being prohibited during certain phases of the 2020 pandemic in the UK, and thus in-person classes not running. There are mixed reports in the scientific literature on the potential impact of attendance at puppy classes on adult dog behaviour. A review by Howell and colleagues in 2015 [38] suggested that attendance at puppy classes did not necessarily confer any benefit, although the authors postulated that the degree of benefit was likely a reflection on how the classes were operated. A large UK-based study of dog owners (*n* = 3897) demonstrated that attendance at puppy classes was associated with a decreased likelihood of dogs displaying aggression towards unfamiliar people both inside and outside the home by 1.4- and 1.6-fold, respectively [39]. In contrast a recent, smaller Spanish study (*n* = 80 dogs) failed to detect an association between attendance at puppy classes and stranger-directed aggression [35]. This smaller study did however find evidence to support an association between reduced non-social fear, touch sensitivity and aggression towards familiar dogs in adult dogs who had attended puppy classes [35]. Another recent investigation of the impact of attendance at puppy class on adult behaviour (*n* = 1023 dogs) found that puppy classes were associated with reduced incidence of aggression, excessive barking, destructive behaviour and compulsive behaviour [36].

In addition to the potential benefits to adult dog behaviour, there is evidence to suggest that attending puppy classes can influence the dog-owner interactions and relationships, with attendance associated with a reduced likelihood of dogs being relinquished in the future [40], and an increased likelihood of owners using reward-based methods involving positive reinforcement and negative punishment [41,42], both of which may be related to adult dog behaviour. Attendance at adult training classes that are based on reward-based methods should be advocated to owners of Pandemic Puppies, who by being thwarted from attending puppy classes may have missed out on both appropriate socialisation experience for their puppies, but also education on appropriate training techniques for their dogs themselves.

Multivariable analysis indicated that younger adult owners and those with children in the household had significantly lower odds of taking their puppy to puppy classes, both before and during the pandemic (2019–2020). Given the importance of puppy classes for owner education as well as dog training [40], and the potential risks posed by inappropriate child-dog interactions in the household [43], encouraging participation in puppy classes in these demographic groups in the future should be a priority for the dog welfare sector. Experienced dog owners were also found to be at lower odds of attending puppy classes, an association that had previously been identified in a study of *n* = 3515 dogs (aged 0 months to >12 years) that were adopted in the UK from Dogs Trust [44].

Owners of small breed/crossbreed puppies were significantly less likely to attend puppy classes compared to medium, large and giant breed/crossbreed puppies, corroborating previous findings that owners of dogs under 20 kg are less likely to engage in dog training [45]. This may be related to owners’ interpretation of what is acceptable dog behaviour, with owners of small breed dogs previously found to be less likely to report behaviours such as pulling on the lead or jumping up as undesirable [46].

Regional variations in puppy class attendance were identified. Other studies have suggested that a variety of socioeconomic factors are associated with the decision for owners to take their puppies to puppy classes or not [47]; however, it is not possible to comment on whether the geographic differences seen in puppy class attendance could be accounted for by socioeconomic status in this study, as these data were not collected. It is possible that some of these differences relate to the local provision of puppy classes, and future initiatives to map availability of these key socialisation experiences may be warranted.

#### 4.1.2. Owner-Led Socialisation Activities

When considering other early-life socialisation and habituation experiences outside of the formal setting of puppy classes, the Pandemic Puppy cohort were exposed to the majority of experiences at the same level as the 2019 puppy cohort, likely reflecting sustained efforts of Pandemic Puppy owners to provide appropriate socialisation for their puppy despite the barriers they faced during the pandemic. It should be noted however, that the number and quality of these exposures were not assessed. Unfortunately, despite these good efforts, some key socialisation activities were still thwarted. Pandemic Puppies were almost three times less likely to have been exposed to visitors to their home before they were aged 16 weeks compared with 2019 puppies, likely due to restrictions imposed on visitors to people’s homes during much of 2020 in the UK [48]. This reduced early socialisation experience is an area of concern in the longer-term, particularly now that restrictions have eased and strangers are more likely to visit Pandemic Puppies’ home environments. It is possible that stranger-related fear or aggression may become an emergent problem for some dogs in the Pandemic Puppy cohort [11] and should be monitored in this population going forward.

### 4.2. Separation-Related Behaviours

Separation-related behaviours (SRBs) are considered the most common behavioural problem in dogs [49] and include a range of undesirable behaviours exhibited in an owner’s absence, with signs including vocalisation, destruction, inappropriate elimination, panting, excessive salivation, vomiting, self-mutilation and repetitive behaviours. Although predisposing factors for this common problem remain poorly understood [49], habituating puppies under the age of 16 weeks to time alone without human contact is considered an important method to reduce the likelihood of development of SRBs [50,51,52]. Whilst the number of owners who had deliberately left their puppies alone under the age of 16 weeks was lower in the Pandemic Puppy cohort (74.6%) compared to the 2019 puppy cohort (81.0%), this could largely be accounted for by some Pandemic Puppies still being under the age of 16 weeks at the time of the survey, and whose owners intended to leave them alone prior to reaching this milestone.

It is of concern that in both 2019 and 2020, around one in six puppies were not deliberately left alone under the age of 16 weeks. For owners within this population intending to leave their dog alone for any length of time in the future (indeed, one in ten Pandemic Puppy owners and one in six 2019 puppy owners planned to leave their dog for over four hours in the future), it is key that owners are made aware of the importance of early life habituation to time alone to avoid future issues with SRBs.

Studies have reported an increase in dogs displaying behaviours indicative of SRBs during the COVID-19 Pandemic in Spain [53] and Italy [54], however a comparable study in the UK demonstrated a significant decrease in dogs exhibiting such behaviours during the first COVID-19 lockdown [55]. This was postulated to be the result of a four-fold increase in the number of owners not leaving their dogs alone during 2020 [21] with the authors citing these changes in dog management having the potential for an increased likelihood of dogs developing SRBs as COVID-19 restrictions ease [20,21]. The incidence of owner-reported SRBs in the Pandemic Puppy cohort should therefore be monitored, and the importance of carefully exposing this population to time alone as COVID-19 restrictions ease emphasised to owners.

### 4.3. Problem Behaviours

‘Problem’ behaviours in dogs are by definition those behaviours which their owners perceive to be undesirable, and as such this is a subjective term with potential inter-owner variation in the desirability, or undesirability, of many normal canine behaviours. The most common ‘problem’ behaviours that Pandemic Puppy owners reported were jumping up at people, mouthing and pulling on their lead. Some of these behaviours are likely age-related, for example, mouthing was reported by over a third of Pandemic Puppy owners, compared to just under a tenth of 2019 puppy owners. Similarly, in a US study, age and breed were associated with mouthing which was reported in 80.0% of dogs under 6 months of age compared to 39.6% of dogs over 12 months of age [56], and had a higher prevalence in Poodles and Poodle crosses [56]. Three of the top five most commonly reported undesirable behaviours in both the Pandemic Puppy and 2019 puppy populations, namely pulling on their lead, jumping up at people and not coming back when called can be considered training issues, perhaps further highlighting the importance of training classes to improve dog behaviour in line with owner expectations, and enhance owner satisfaction with their dogs [57]. These same three behaviours were those most commonly reported in a recent survey of the UK ‘Generation Pup’ cohort (*n* = 1111) with non-attendance at a puppy class one of the risk factors in owners not reporting these behaviours as undesirable [46]. As such, the true incidence of these behaviours in both the 2019 puppy and Pandemic Puppy cohorts could be under-reported, given that this study only explored behaviours that owners considered undesirable.

### 4.4. Preventative Healthcare and Early-Life Health

Early life healthcare provisions for puppies were largely maintained during the pandemic. Levels of preventative and other healthcare activities including registration with a veterinary surgeon, insurance, use of flea and de-worming treatments and microchipping, did not significantly differ between 2019 puppies and Pandemic Puppies. Any significant differences seen in other preventative healthcare provisions such as vaccination and neutering appeared to simply be a reflection of the lower age of the Pandemic Puppy cohort (with some dogs still <16 weeks of age at the time of survey) and/or their owners having not yet made a decision. This suggests that by the time of the survey in November-December 2020, despite COVID-19 restrictions limiting access to preventative veterinary healthcare in the UK [58], levels of vaccination, neutering and parasite control had returned to pre-pandemic levels, as noted in the 2021 PDSA Paw Report [17].

The early-life health profile of Pandemic Puppies did not markedly differ from 2019 puppies. For both cohorts, the health disorders most commonly reported soon after a puppy was brought home were enteropathy, parasite infestation, skin (cutaneous) disorder and ophthalmological disorder findings. The only identifiable difference between cohorts at this age was a significantly higher prevalence of skin disorders and parasite infestation in Pandemic Puppies compared to 2019 puppies. Using VetCompass™ disorder terms, the top three most prevalent disorders in the UK dog population have previously included enteropathy and dermatological disorders, and thus the disorder burden of these puppy populations appears in line with that of the general UK dog population [22,59]. Parasite infestation featuring highly in both cohorts in the current study is likely a reflection of owners reporting puppy health soon after acquisition (at <16 weeks of age), whereas previous studies on the epidemiology of canine health in the UK have included dogs of all life stages [22,59]. The significantly higher prevalence of parasite infestation in the Pandemic Puppy cohort could be indicative of Pandemic Puppies being more likely to be acquired from lower welfare sources with poor husbandry and preventative healthcare, or a reflection of temporary restrictions in access to preventative veterinary healthcare in 2020 prior to this survey [58,60]. However, as these data were self-reported by owners rather than extracted from veterinary records, the increase in skin disorders in the Pandemic Puppy population could be a reflection of owners being more likely to notice their puppies itching as a result of increased time spent with them during the pandemic, or could simply reflect a recall bias effect upon 2019 owners given the longer timescale between assessment age and time of report.

### 4.5. Early Provisions and Advice Offered by Breeders

Despite the legal requirement for breeders to microchip their puppies prior to sale at (a minimum of) 8 weeks in the UK [61,62,63,64], only 93.2% of Pandemic Puppies and 92.4% of 2019 puppies were reported as microchipped prior to being taken home, and only 92.1% of Pandemic Puppy owners and 92.7% of 2019 puppy owners were provided with their puppy’s microchip details. As such, greater awareness and/or enforcement of this requirement is needed for UK breeders, but also greater awareness amongst puppy buyers of this potential ‘red flag’ for illegal sales, where breeders are not being compliant with this legislation.

The proportion of puppies sold with a Pet Passport at the time of acquisition was significantly higher in the Pandemic Puppy population (7.1%) than in the 2019 puppy population (4.1%). Sales of puppies with passports has been cited as a potential ‘red flag’ for puppies having been imported illegally via puppy smugglers [65], with canine welfare organisations reporting concerns at an over 100% increase in the number of puppies imported during 2020 compared to before the pandemic (2019) [6,66]. It appears that the demand for puppies during the pandemic outstripped supply from legitimate, welfare-conscious sources, leading to some owners unknowingly supporting the growing European puppy import trade [67]. This is of high welfare concern, given the potentially poor (unseen) conditions in which puppies may have been raised in intensive breeding establishments, compounded by travelling long distances at a young age without their mothers. Indeed, investigations have identified Eastern Europe as a common origin of imported puppies, with journeys to the UK potentially taking several days by road. As highlighted by Maher and Wyatt [67], puppy farming being legal in Eastern Europe presents a lucrative business model to mass produce puppies that are worth in excess of £1000 in a location that is relatively cheap (costing as little as £25 per puppy) [67]. Both the regulations for commercial transport of puppies (the Balai Directive) and the non-commercial Pet Travel Regulations require puppies to be a minimum of 15 weeks old at importation, for the purposes of rabies control. A total of 87.4% of puppies sold with passports in 2020 were under 13 weeks at sale, potentially contravening this legislation. Increasing owner awareness of the welfare implications of buying an imported puppy is of increasing importance to avoid this becoming an accepted source of puppies in the UK market [6,66], threatening canine health and welfare but also the control of infectious diseases. Valuable resources for veterinary surgeons suspecting illegal importation of puppies have been developed so suspicions can be raised to appropriate authorities to tackle this illegal trade [68].

### 4.6. Breed Demographics

There was a shift towards preferential purchasing of designer crossbred dogs over purebred dogs in the Pandemic Puppy cohort, with particular pandemic favorites including the Cockapoo, Labradoodle and Cavapoo. A similar trend has been reported internationally; for example, analysis of insurance policies taken out following the start of the pandemic revealed that the Cavapoo was the most popular of all breeds and crossbreeds purchased in Australia during this time [69], whilst a popular online pet sales platform in the UK reported the Cavapoo as the dog type most in demand during the 2020 phase of the COVID-19 pandemic, closely followed by the Cockapoo [70]. The increase in popularity of designer breeds during the pandemic could reflect the shifting demographic of Pandemic Puppy owners, already documented in this population [1]. Pandemic Puppy owners were more likely to be first-time dog owners with children in their household, often with children aged 5–10 years of age, who sought a breed/crossbreed that would fulfil lifestyle factors such as being a suitable size for their lifestyle, good with children, easy to train and hypoallergenic [1]. The belief that designer crossbreeds are ‘hypoallergenic’ is a popular misconception [71], and could potentially lead to unmet owner expectations and allergies if the crossbreed was explicitly selected for this reason.

The most popular purebred breeds in the current study differ to Kennel Club registrations during the 2020 period of the COVID-19 pandemic in the UK [72]. Although four of the purebreds in the top 10 of the Pandemic Puppies’ cohort were also in the top seven of all Kennel Club registrations during 2020—namely the Labrador Retriever, Cocker Spaniel, Miniature Smooth Dachshund and Golden Retriever, notably there were fewer brachycephalic puppies represented for either year in the current study. It has however been reported that the most popular brachycephalic breed, the French Bulldog, was only the 18th most popular dog purchased on a popular UK internet selling platform during the 2020 phase of the pandemic [70]. An apparent underrepresentation of French Bulldogs, English Bulldogs and Pugs relative to Kennel Club registration statistics has also been reported in the Generation Pup cohort of *n* = 3726 puppies from the UK [73] which also reported the designer crossbreeds Cockapoo, Labradoodle and Sprocker, as the three most popular crossbreeds in the study [73]. Reasons for this underrepresentation are unknown, but may reflect breed-related differences in owner propensity to engage in canine health and welfare studies, with demographic differences in brachycephalic vs. non-brachycephalic dog owners previously documented [74]. An overrepresentation of Golden Retrievers and Border Collies relative to Kennel Club registration statistics was noted in both the 2019 puppy and Pandemic Puppy cohorts, and has previously been reported in the Generation Pup cohort [73]. This would suggest that Kennel Club registration statistics do not reflect the relative popularity of different purebred dogs in the overall UK dog population; however, without obligatory registration of UK dogs to a centralised register (as seen in other European countries, such as Denmark) it is not possible to estimate how widely these populations differ, or how representative study samples such as the Pandemic Puppy cohort are.

### 4.7. Limitations and Future Research

The data presented on health and socialisation experiences soon after acquisition/under the age of 16 weeks may be subject to recall bias, particularly in the 2019 cohort, given the longer timescale between events and time of recall. This effect did not appear explicitly problematic, with few owners of 2019 puppies answering ‘I’m not sure/can’t remember’ regarding their dogs’ early life experiences. In addition, due to social desirability bias, it is possible that owners who were aware that their puppy’s socialisation experiences and/or health were less than ideal may not have responded to the survey.

Future longitudinal study of the 2020 Pandemic Puppy cohort will examine key outcomes including health, behaviour, the dog-owner bond and relinquishment risk to identify risks and opportunities for the welfare of this unique population as they age.

Given that the trend for increased interest in puppy acquisition during the pandemic occurred internationally [5], research is needed to explore whether the same early-life deficits occurred for puppies born outside of the UK. This is particularly pressing in countries where the strictest lockdown measures were employed to restrict the transmission of the SARS-CoV-2 virus, given that these conditions were most likely to thwart the comprehensive socialisation/habituation of puppies. These data will have implications for international canine welfare, but also public health risk, given the documented increase in dog bites during the Pandemic in several countries including the UK [75], USA [76] and Italy [77], which may continue post-pandemic if dogs are inadequately socialised to visiting strangers, for example. Understanding the early lives of dogs born during this unprecedented period will help countries to identify the presence and nature of socialisation/habituation deficits and thus the most appropriate measures to promote good behaviour and dog-owner relationships in the future.

## 5. Conclusions

Despite the drastic lifestyle changes imposed upon owners by social restrictions, the proportion of Pandemic Puppies exposed to many important early life socialisation and habituation experiences under the age of 16 weeks did not significantly reduce from pre-pandemic levels. However, this study identified reduced levels of attendance at puppy classes and a reduction in exposure to visitors to the home amongst the Pandemic Puppy cohort during their critical developmental period, which has the potential to lead to the development of behavioural issues in the future. Understanding these differences will be vital to helping animal welfare organisations, veterinary professionals and animal behaviour professionals better support this unique population of dogs and owners in the future to avoid or address poor outcomes which could lead to increased risks of relinquishment. For example, creation of bespoke public-facing resources from animal behavioural professionals to manage and ameliorate the effects of inadequate socialisation is needed to supplement provision of individual behaviour support, which may be stretched by the growing canine population. In addition, increased surveillance from veterinary professionals regarding emerging health and/or behavioural concerns will help to identify pandemic-related changes as this vulnerable population ages. This study also identified concerning changes in provenance and demographics of puppies, with a surge in designer crossbreed puppies and puppies sold with a Pet Passport during the pandemic. Educating buyers on how best to avoid inadvertently supporting the illegal puppy trade and re-thinking the domestic supply of dogs from higher welfare sources for the UK puppy market is of high priority to protect canine welfare.

## Figures and Tables

**Table 1:** Most popular individual breeds/crossbreeds in the 2019 puppy (*n* = 1147) and Pandemic Puppy (*n* = 4358) cohorts. Crossbred dogs were defined as mixed breeds of unknown origin, or crosses who were not reported using a breed-indicative designer crossbreed name, e.g., Spaniel Cross. The breeds with the greatest frequency, which formed the top ten most popular breeds for each of the 2019 puppy and Pandemic Puppy cohorts, are shown along with their ranking (1 = most popular) for each year. The *p* value reflects the probability of a difference in popularity for the breed/crossbreed between 2019 to 2020. Significant differences are emboldened.

Breed/Crossbreed	2019			2020			Statistics		
	*n*	%	Rank	*n*	%	Rank	% Change 2019 to 2020	*X* ^2^	*p* Value
Labrador Retriever	134	11.7	1	429	9.9	1	−15.4	3.34	0.067
Cocker Spaniel	81	7.1	2	324	7.4	3	+4.2	0.19	0.667
**Cockapoo**	**72**	**6.3**	**3**	**362**	**8.3**	**2**	**+31.7**	**5.15**	**0.023**
Miniature Smooth-Haired Dachshund	64	5.6	4	188	4.4	4	−21.4	3.33	0.068
**Border Collie**	**58**	**5.1**	**5**	**150**	**3.4**	**5**	**−33.3**	**6.51**	**0.011**
**Golden Retriever**	**52**	**4.6**	**6**	**120**	**2.8**	**9**	**−39.1**	**9.51**	**0.002**
Border Terrier	42	3.7	7	140	3.2	7	−13.5	0.57	0.449
Crossbreed	29	2.5	8	149	3.5	6	+40.0	2.30	0.129
German Shepherd Dog	27	2.4	9	86	2.0	12	−16.7	0.65	0.419
English Springer Spaniel	24	2.1	10	96	2.2	11	+4.8	0.02	0.820
Labradoodle	23	2.0	11	127	2.9	8	+45.0	2.83	0.092
Cavapoo	20	1.7	12	111	2.6	10	+52.9	2.52	0.112

**Table 2:** Provision of preventative veterinary care, advice and offers of ongoing relationships from breeders to owners of Pandemic Puppies and owners of 2019 puppies (a) prior to taking puppies home; (b) at collection; and (c) at any time point. Significant differences between the two cohorts are emboldened. * Non-commercial Pet Travel Regulations in the UK require puppies to have a Pet Passport and be a minimum of 15 weeks old at importation, for the purposes of rabies control.

Timepoint	Breeder Provision	Acquisition Year		Statistics	
		2019%	2020%	*X^2^*	*p* Value
**Prior to bringing puppy home** (Total *n* = 5168; 2019 *n* = 1063, 2020 *n* = 4035)	Microchip	92.4	93.2	0.84	0.360
	Worming treatment	89.8	89.6	0.05	0.831
	**Health check by a veterinary surgeon**	**91.3**	87.4	12.07	**<0.001**
	Flea treatment	69.3	68.6	0.18	0.669
	First vaccinations	67.0	67.4	0.06	0.802
	**Second vaccinations**	**8.4**	6.4	5.05	**0.025**
**When puppy was collected** (Total *n* = 4368; 2019 *n* = 900, 2020 *n* = 3468)	Puppy’s microchip details	92.7	92.1	0.62	0.891
	Puppy’s vaccinations record	69.0	68.8	6.48	0.090
	**Feeding guidance in writing**	**68.8**	61.9	32.91	**<0.001**
	**Copy of puppy’s pedigree**	**61.7**	47.3	70.10	**<0.001**
	**Kennel Club change of ownership form**	**59.4**	43.6	76.90	**<0.001**
	Puppy’s passport *	4.1	**7.1**	45.51	**<0.001**
**At any point in time** (*n* = 4462; 2019 = 921, 2020 = 3541)	**Advice on diet**	61.6	**76.9**	88.76	**<0.001**
	**Advice on your puppy’s health**	49.2	**58.0**	23.25	**<0.001**
	**Advice on training/behaviour**	42.1	**50.8**	17.24	**<0.001**
	The option to return puppy to them in the future for any reason	50.1	47.3	2.22	0.136
	**Advice on exercise regime**	32.5	**37.8**	8.91	**0.003**
	**None of the above**	**21.5**	14.4	27.57	**<0.001**
	**The option to board puppy with them when on holiday**	**17.0**	12.0	16.19	**<0.001**

**Table 3:** Age when taken home for 2019 puppies (*n* = 884) and Pandemic Puppies (*n* = 3430) sold under 16 weeks with a Pet Passport.

Puppies Sold with a Pet Passport (*n* = 4314)	Acquisition Year	
	2019% (*n* = 884)	2020% (*n* = 3430)
13 to 16 weeks old	27.8	12.6
11 to 12 weeks old	2.8	5.7
9 to 10 weeks old	33.3	25.2
7 to 8 weeks old	36.1	56.5
Under 6 weeks old	0.0	0.0
I’m not sure/I can’t remember	0.0	0.0

**Table 4:** Owner reports of health issues in 2019 puppies (*n* = 1148) and Pandemic Puppies (*n* = 4369) soon after being brought home. Disorders listed are VetCompass™ disorder terms [23]. * The responses to the options for the question were categorised here in addition to free-text responses. Significant differences between the two cohorts are emboldened.

Soon after You Brought Your Puppy Home, Did You Notice Any of the Following? (*n* = 5571)	Acquisition Year		Statistics	
	2019% (*n* = 1148)	2020% (*n* = 4369)	*X^2^*	*p* Value
Enteropathy *	10.2	9.9	0.09	0.760
Parasite infestation *	2.1	**4.7**	5.27	**0.022**
Skin (cutaneous) disorder *	2.2	**4.3**	10.88	**<0.001**
Ophthalmological disorder *	2.6	2.4	0.21	0.648
Upper respiratory tract disorder *	0.5	0.7	0.59	0.444
Ear (aural) disorder	0.4	0.2	1.89	0.169
Undesirable behaviour disorder	0.3	0.2	0.13	0.720
Thin/underweight	0.2	0.1	0.57	0.450
Traumatic injury	0.3	0.1	3.11	0.078
Musculoskeletal disorder	0.0	0.1	1.32	0.251
Polyuria/Polydipsia	0.1	0.1	0.04	0.836
Anal sac disorder	0.0	0.0	0.53	0.468
Haematopoietic system disorder	0.1	0.0	3.81	0.051
Oral cavity (mouth) disorder	0.0	0.0	0.26	0.608
Vascular disorder	0.0	0.0	0.26	0.608
Female reproductive abnormality	0.1	0.0	0.29	0.593
Lower respiratory tract disorder	0.0	0.0	0.26	0.608
Hearing impaired/deafness	0.0	0.0	0.26	0.608
Heart (cardiac) disease	0.0	0.0	0.53	0.468
Collapsed	0.0	0.0	0.26	0.608
Hernia	0.0	0.0	0.53	0.468
Lethargy	0.0	0.0	0.53	0.468
Mass/lump/swelling	0.0	0.0	0.26	0.608
Appetite disorder	0.1	0.0	1.04	0.309
Tail disorder	0.0	0.0	0.26	0.608
Urinary system disorder	0.1	0.0	0.29	0.593
Drug therapy adverse reaction	0.0	0.0	0.63	0.468
Abdominal disorder	0.0	0.0	0.26	0.608
Foreign body	0.0	0.0	0.26	0.608
Complication associated with clinical care procedure	0.0	0.0	0.26	0.608

**Table 5:** Insurance status of 2019 puppies (*n* = 1140) and Pandemic Puppies (*n* = 4352) as reported by owners at the time of the survey in response to the question “Is your puppy/dog insured”. The answer options listed represent all the choices available to respondents.

Is Your Puppy/Dog Insured? (*n* = 5439)	Acquisition Year	
	2019% (*n* = 1140)	2020% (*n* = 4352)
Yes	83.5	84.0
No, and I do not plan to insure them	10.7	7.3
No, but I plan to insure them in the future	3.2	7.0
No, they were insured but I have since cancelled or did not renew their policy	2.5	1.7
No, I have never heard of pet insurance	0.1	0.0

**Table 6:** Vaccination status of 2019 puppies (*n* = 1073) and Pandemic Puppies (*n* = 4099) as reported by the owners at the time of the survey in response to the question “Has your puppy/dog been vaccinated, or do you plan to in the future”. The answer options listed represent all the choices available to respondents.

Has Your Puppy/Dog Been Vaccinated, or Do You Plan to in the Future? (*n* = 5172)	Acquisition Year	
	2019% (*n* = 1073)	2020% (*n* = 4099)
Yes—first and second vaccinations	92.7	82.5
Yes—just their first vaccinations	6.8	15.2
No—not yet, but I plan to in the future	0.2	1.9
No—I have chosen not to vaccinate my puppy/dog and don’t plan to in the future	0.3	0.2
No—not yet, I haven’t decided	0.0	0.2

**Table 7:** Neuter status of 2019 puppies (*n* = 1053) and Pandemic Puppies (*n* = 4072) as reported by their owners at the time of the survey in response to the question “Has your puppy/dog been neutered, or do you plan to in the future”. The answer options listed represent all the choices available to respondents.

Has Your Puppy/Dog Been Neutered, or Do you Plan to Have Them Neutered in the Future? (*n* = 5098)	Acquisition Year	
	2019% (*n* = 1053)	2020% (*n* = 4072)
No, but I intend to have them neutered when they are older	21.4	45.4
No, not yet, I haven’t decided	14.2	21.7
No, but I do not plan to breed from them	11.2	7.2
No, because I plan to breed from them	10.3	6.1
Yes, aged under 6 months	3.6	2.9
Yes, aged over 6 months	39.4	16.6

**Table 8:** Proportion of 2019 puppies (*n* = 1052) and Pandemic Puppies (*n* = 4049) deliberately left alone under the age of 16 weeks. The answer options listed represent all the choices available to respondents.

Whilst Your Puppy Was Under 16 Weeks of Age, Did You Deliberately Leave Them Alone for Any Period of Time to Get Them Used to Being Left Alone? (*n* = 5101)	Acquisition Year	
	2019% (*n* = 1052)	2020% (*n* = 4049)
Yes	81.0	74.6
No	16.6	15.9
No, not as yet but I plan to before my puppy is 16 weeks old (if applicable)	0.1	9.0
I can’t remember	2.3	0.5

**Table 9:** Comparison of owner-reported socialisation experiences under the age of 16 weeks of 2019 puppies and Pandemic Puppies in response to the question “Did your puppy/dog encounter any of the following experiences between you buying them and reaching 16 weeks of age”. Sample size varied for each experience and are therefore shown in brackets. The response options listed represent all the choices available to respondents. Significant differences between the two cohorts are emboldened.

Experience (Total; 2019, 2020)	Response	Acquisition Year		Statistics
		2019%	2020%	
**Travelling in a car** (total *n* = 4461; 2019 *n* = 921, 2020 = 3540)	Yes	98.9	98.6	*X*^2^ = 5.80 *p* < 0.122
	No	1.1	0.8	
	I’m not sure/can’t remember	0.0	0.0	
	No, not as yet but I plan to before my puppy is 16 weeks old (if applicable)	0.0	0.5	
**Meet any people from outside your household** (total *n* = 4466; 2019 *n* = 922, 2020 *n* = 3544)	Yes	93.8	90.5	***X*** **^2^** **= 35.89** ***p*** **< 0.001**
	No	4.8	5.6	
	I’m not sure/can’t remember	1.4	0.7	
	No, not as yet but I plan to before my puppy is 16 weeks old (if applicable)	0.0	3.2	
**Visitors to their home** (total *n* = 4449; 2019 *n* = 920, 2020 *n* = 3529)	Yes	94.5	81.8	***X*** **^2^** **= 103.05** ***p*** **< 0.001**
	No	4.6	12.8	
	I’m not sure/can’t remember	1.0	0.7	
	No, not as yet but I plan to before my puppy is 16 weeks old (if applicable)	0.0	4.8	
**Walking in a public space (i.e., outside of your home/ garden)** (total *n* = 4459; 2019 *n* = 921, 2020 *n* = 3538)	Yes	87.5	78.3	***X*** **^2^** **= 151.27** ***p*** **< 0.001**
	No	10.7	9.4	
	I’m not sure/can’t remember	1.7	0.2	
	No, not as yet but I plan to before my puppy is 16 weeks old (if applicable)	0.0	12.1	
**Walking near traffic** (total *n* = 4438; 2019 *n* = 918, 2020 *n* = 3520)	Yes	85.0	78.0	***X*** **^2^** **= 131.16** ***p*** **< 0.001**
	No	13.1	10.9	
	I’m not sure/can’t remember	2.0	0.4	
	No, not as yet but I plan to before my puppy is 16 weeks old (if applicable)	0.0	10.8	
**Meet any dogs from outside your household** (total *n* = 4466; 2019 *n* = 922, 2020 *n* = 3544)	Yes	80.4	76.3	***X*** **^2^** **= 86.28** ***p*** **< 0.001**
	No	18.0	14.8	
	I’m not sure/can’t remember	1.6	0.8	
	No, not as yet but I plan to before my puppy is 16 weeks old (if applicable)	0.0	8.1	
**Fireworks** (total *n* = 4394; 2019 *n* = 904, 2020 *n* = 3490)	Yes	31.7	44.4	***X*** **^2^** **= 132.01** ***p*** **< 0.001**
	No	61.3	50.8	
	I’m not sure/can’t remember	7.0	1.9	
	No, not as yet but I plan to before my puppy is 16 weeks old (if applicable)	0.0	2.9	
**Thunderstorms** (total *n* = 4362; 2019 *n* = 900, 2020 *n* = 3462)	Yes	28.0	30.9	***X*** **^2^** **= 294.31** ***p*** **< 0.001**
	No	43.6	55.9	
	I’m not sure/can’t remember	28.4	8.4	
	No, not as yet but I plan to before my puppy is 16 weeks old (if applicable)	0.0	4.8	
**Dog groomer** (total *n* = 4376; 2019 *n* = 898, 2020 *n* = 3478)	Yes	19.9	20.2	***X*** **^2^** **= 99.31** ***p*** **< 0.001**
	No	78.3	70.8	
	I’m not sure/can’t remember	1.8	0.4	
	No, not as yet but I plan to before my puppy is 16 weeks old (if applicable)	0.0	8.5	

**Table 10:** Proportion of 2019 puppies (*n* = 1053) and Pandemic Puppies (*n* = 4047) attending puppy classes under the age of 16 weeks. * Response options shown here were formulated via qualitative content analysis of the free-text option “No, other (please describe here)”.

Did You or Someone in Your Household Attend Any Puppy Classes with Your Puppy before They Were 16 Weeks Old? (*n* = 5100)	Acquisition Year	
	2019% (*n* = 1053)	2020% (*n* = 4047)
Yes, in-person puppy classes	67.9	28.9
No, I wanted to but there weren’t any classes running	8.3	28.9
No, I do not intend to	19.6	17.7
No, not as yet but I plan to before my puppy is 16 weeks old (if applicable)	0.0	16.6
Yes, online puppy classes	1.0	6.7
* No, I chose not to because I am a dog professional	1.3	0.9
* No, I was unable to attend < 16 weeks due to my puppy’s circumstances (e.g., poor health, acquired close to 16 weeks)	1.3	0.3
* No, my puppy died aged <16 weeks	0.0	0.1
* No, I was unable to attend classes while my puppy was <16 weeks due to my own circumstances (e.g., health, work commitments)	0.7	0.0

**Table 11:** Final multivariable logistic regression model for variables examined for association with attendance at either in-person or online puppy training classes under the age of 16 weeks (*n* = 5117: 2019 *n* = 1054; 2020 *n* = 4063). Significant associations are emboldened. * Confidence interval.

	Variable	Category	Odds Ratio	95% CI *	*p* Value
**Year**	Year of acquisition	2019		Reference	
		**2020**	**0.23**	**0.20 to 0.27**	**<0.001**
**Dog demographics**	Breed designation	Crossbred		Reference	
		Purebred	1.39	0.97 to 1.98	0.074
		Designer Crossbred	1.35	0.94 to 1.96	0.109
	Typical adult bodyweight	**≤10 kg**	**0.65**	**0.55 to 0.76**	**<0.001**
		10 to <20 kg		Reference	
		20 to <30 kg	1.07	0.91 to 1.26	0.430
		30 to <40 kg	1.06	0.88 to 1.28	0.557
		≥40 kg	1.26	0.85 to 1.87	0.255
	Dog sex	Male		Reference	
		Female	0.99	0.88 to 1.12	0.867
	Neutered	No		Reference	
		Yes	1.12	0.97 to 1.29	0.120
**Owner demographics**	Owner age	**18 to 24 years old**	**0.61**	**0.46 to 0.81**	**<0.001**
		25 to 34 years old		Reference	
		**35 to 44 years old**	**1.23**	**1.01 to 1.50**	**0.037**
		**45 to 54 years old**	**1.21**	**1.01 to 1.46**	**0.045**
		55 to 64 years old	1.04	0.85 to 1.28	0.709
		65 to 74 years old	0.87	0.66 to 1.15	0.334
		75 years or older	1.30	0.67 to 2.49	0.436
	Owner gender	Female		Reference	
		**Male**	**0.75**	**0.60 to 0.92**	**0.007**
		Other	0.78	0.23 to 2.60	0.685
	Live with children	No		Reference	
		**Yes**	**0.86**	**0.74 to 0.99**	**0.042**
	Region	Scotland		Reference	
		**Northern Ireland**	**0.32**	**0.13 to 0.77**	**0.011**
		Wales	0.96	0.66 to 1.41	0.840
		**East of England**	**2.12**	**1.63 to 2.75**	**<0.001**
		East Midlands	1.37	0.99 to 1.88	0.055
		**London**	**1.59**	**1.19 to 2.11**	**0.001**
		North East	1.34	0.93 to 1.94	0.121
		North West	1.19	0.88 to 1.63	0.261
		**South East**	**1.74**	**1.35 to 2.25**	**<0.001**
		**South West**	**1.67**	**1.26 to 2.20**	**<0.001**
		**West Midlands**	**1.42**	**1.04 to 1.93**	**0.028**
		Yorkshire and The Humber	1.22	0.90 to 1.66	0.202
**Dog owning experience**	Previously owned a dog	No		Reference	
		**Yes**	**0.59**	**0.51 to 0.68**	**<0.001**

**Table 12:** Comparison of owner reported undesirable behaviours in 2019 puppies/dogs (*n* = 915) and Pandemic Puppies (*n* = 3511) at the time of the survey. The answer options listed represent all the choices available to respondents.

Does Your Puppy/Dog Currently Show Any of the Following Behaviours that You/Your Household Find Problematic? (*n* = 4426)	Acquisition Year	
	2019% (*n* = 915)	2020% (*n* = 3511)
Jumping up at people	30.3	36.1
Mouthing	9.7	33.1
Pulling on their lead	38.6	31.0
Clinginess (e.g., following you, sitting close)	19.8	23.7
Not coming back when called	21.7	16.3
Barking or howling when left alone	10.9	15.8
Chasing, e.g., cats, wildlife	24.9	10.6
Toileting (weeing or pooing) in the house when left alone	4.4	9.1
Barking at other dogs	16.9	8.1
Anxiety/fear around unfamiliar people	10.1	6.3
Fear of loud sounds (e.g., fireworks, thunderstorms)	8.3	4.9
Anxiety/fear around other dogs	6.8	4.3
Being destructive when left alone	5.2	4.3
Guarding of food, toys, or other items	4.9	2.8
Aggression towards people in your household (including you)	0.9	1.0
Aggression towards unfamiliar people	2.5	0.6
Aggression towards other dogs	3.5	0.5
Anxiety/fear around people in your household (including you)	0.5	0.3
Not applicable – I no longer have my puppy	0.1	0.3
None of the above	23.0	22.9

**Table 13:** Comparison of owner reported relinquishment or considered relinquishment between 2019 puppies (*n* = 1050) and Pandemic Puppies (*n* = 4044). The answer options listed represent all the choices available to respondents.

Have You Considered, or Have You Needed to, Rehome Your Puppy/Dog Since You Acquired Them? (*n* = 5094)	Acquisition Year	
	2019% (*n* = 1050)	2020% (*n* = 4044)
I still have my puppy/dog and have not considered rehoming them	98.6	98.3
I still have my puppy/dog, but I have considered, or I am currently considering rehoming them	0.9	1.2
Not applicable—my puppy/dog has passed away	0.1	0.2
Not applicable—my puppy/dog was put to sleep	0.2	0.1
I have rehomed my puppy/dog to another person/family	0.2	0.0

## Data Availability

The data presented in this study are available on request from the corresponding author.

## Supplementary Materials

The following are available online at https://www.mdpi.com/article/10.3390/ani12050629/s1, File S1: Questionnaire, File S2: Qualitative Coding Framework—Puppy Classes, File S3: Coding Framework VetCompass™ Disorder terms.
