# Supplementary material for: Pandemic Puppies: Demographic Characteristics, Health and Early Life Experiences of Puppies Acquired during the 2020 Phase of the COVID-19 Pandemic in the UK
Source: Animals (Basel). 2022 Mar 2;12(5):629. doi: 10.3390/ani12050629 (PMC8909199; doi:10.3390/ani12050629)
Supplement: Supplementary file 1 [file animals-12-00629-s001.zip › animals-1606453-supplementary/Brand et al File S1 Questionnaire.pdf]

CONSENT QUESTIONS AND INCLUSION CRITERIA:

Q1

I confirm that:

[SURVEY LOGIC – If no to any question take to disqualification page]

1. I am over 18 years of age
2. I am a resident of the UK
3. I have read and understood the above information and give consent for my answers to be used for this research study and any resulting publications
4. I brought a puppy home aged under 16 weeks during 2019 OR 2020

ESSENTIAL QUESTIONS (ALL OWNERS):

Q2

When did you bring your puppy home?

Drop down month list

Drop down year list (2019 & 2020)

Q3

Please select the breed or crossbreed of dog you are answering for.

Drop down list

Not on the list (please specify) **[free text]**

Q4

Is your puppy/dog registered with The Kennel Club?

Yes

Not applicable – crossbreed

Not applicable – breed not registered with The Kennel Club

I'm not sure

No

No – registered with another canine registration body, e.g. a working dog registry (please specify) **[free text]**

Q5

What is your puppy's/dog's date of birth? Please enter in the format DD/MM/YY

N.B. If you are unsure, please leave relevant part as '00'

Date text field

Q6

<u>What sex is your puppy/dog?</u>

Male

Female

Q7

<u>Is your puppy/dog insured?</u>

Yes

No – and I do not plan to insure them

No – but I plan to insure them in the future

No – they were insured but I have since cancelled or did not renew their policy

No – I have never heard of pet insurance

No – other **[free text]**

Q8

<u>What were the main reasons your household wanted to acquire a dog?</u>

<u>Please select **all options** that apply</u> **[RANDOMISE LIST FOR EACH SURVEY TO AVOID BIAS]**

Companionship for myself

Companionship for my children

Companionship for other adult(s) in my household

Companionship for my other dog(s)

To keep me/my family busy

To encourage myself/my family to walk and exercise

To improve my/my family's mental health

Due to the loss of a previous dog in my household

As a working dog for a specific role (e.g. gundog, security, sniffer/tracking, herding, medical detection, assistance/therapy dog)

Other reason not listed above **[free text]**

Q9

<u>Did you or your household carry out any research into owning dog and/or which breed/crossbreed to buy before you purchased your puppy?</u>

1. No **[SURVEY LOGIC – skip question 10]**
2. No – but I am already an experienced dog owner **[SURVEY LOGIC – skip question 10]**
3. Yes – please describe in your own words the research you carried out **[free text]**

Q10

[SURVEY LOGIC – If answering yes to option 3 above (Q9)]

What sources of information did you or your household use when researching dog ownership and/or which breed/crossbreed to buy prior to buying your puppy?

Please select **all options** that apply **[FLIP CHOICES FOR EACH SURVEY TO AVOID BIAS]**

None of these options

The Kennel Club website

An animal charity website, e.g. Dogs Trust, RSPCA, PDSA, etc.

A breed/crossbreed-specific online resource (e.g. website/forum)

Social media sites, e.g. Facebook, Instagram

Book(s)

Dog-specific magazine(s)

My veterinary professional (e.g. veterinary surgeon, veterinary nurse)

Talking to friends or family who own or had owned a dog

Talking to a dog breeder

I can't remember

Other (please specify) **[free text]**

Q11

What characteristics were you looking for in a dog when selecting a particular breed/crossbreed to buy? Please select **all options** that apply **[FLIP CHOICES FOR EACH SURVEY TO AVOID BIAS]**

I've owned this breed or crossbreed before
I grew up with or had childhood experiences with this breed/crossbreed
Friends or family currently own this breed/crossbreed
Affordable purchase cost of puppies
Affordable cost of upkeep
Appearance/looks
Low grooming needs
Low exercise requirements
Good with children
Good companion
Size suited to my lifestyle
Generally healthy breed/crossbreed
Popularity of the breed/crossbreed
Working ability of the breed/crossbreed
Long life expectancy
Exercise encouragement
Celebrity/Influencer endorsement/ownership
Hypoallergenic
Easy to train
None of these options – I did not have any specific characteristics I was looking for
Other (please tell us here) **[free text]**

Q12

How long after you/your household decided to look for a puppy did you bring your puppy/dog home?

Less than 1 week
Between 1 week-1 month
1 month-6 months
>6 months
I don't remember

Q13

Was the breed or crossbreed of your puppy/dog your first choice?

1. Yes, my/our puppy/dog is the breed/crossbreed that was my/our first choice **[SURVEY LOGIC – skip question 14]**
2. No, I/we could not find a seller that had puppies available at the time for my/our first choice breed/crossbreed
3. No, I/we could not find a breeder I/we felt happy buying a puppy from for my/our first choice breed/crossbreed
4. No, puppies of my/our first choice breed/crossbreed were too expensive
5. No, puppies of my/our first choice breed/crossbreed were too far away
6. Other (please specify) **[free text]**

Q14

[SURVEY LOGIC – If answering anything except for option 1 above (Q13)]

If your puppy/dog is not your first choice of breed or crossbreed, please let us know which breed/crossbreed was your first choice below.

[free text]

Q15

How did you find the breeder of your puppy/dog?

Please select **all options** that apply **[RANDOMISE LIST FOR EACH SURVEY TO AVOID BIAS]**

A general selling website, e.g. FreeAds, Gumtree, Preloved

An animal specific selling website, e.g. Pets4Homes, Champdogs

The Kennel Club website 'Find A Puppy' search

The breeder's website

The breeder's social media account

A social media breed/crossbreed-specific group

Local newspaper advert

Dog specific magazine(s)/newspaper(s)

An advert in a local shop

I already knew the breeder (e.g. colleague, friends, family, repeat purchase)

Recommendation from a friend

Other (please specify) **[free text]**

Q16

What characteristics were you looking for in a breeder?

Please select **all options** that apply **[RANDOMISE LIST FOR EACH SURVEY TO AVOID BIAS]**

Availability of the breed I wanted

Availability of puppies at the time I wanted

Bred the colour of the breed/crossbreed I wanted to purchase

Reasonably priced puppies

Lived within the distance I was willing to travel

Good communication with me

They performed health tests for the breed/crossbreed I wanted

Someone I felt was trustworthy

Someone I felt cared for their dogs

They would allow me to see the puppies' mother

They would allow me to see the puppies' father

They registered their puppies with The Kennel Club

The dogs they bred from had been awarded prizes at dog shows

A member of the Kennel Club Assured Breeder Scheme

Other (please specify) **[free text]**

Q17

Did your breeder question you about your suitability as a dog owner before they agreed to sell you your puppy/dog?

Yes

No

I don't remember

Q18

How much did you pay in total to purchase your puppy/dog?

N.B. Please only include the price of your puppy and not any associated purchases, e.g. food, collar, bowls, etc.

£ (please state a whole number in pounds, do not include the pound sign) **[Number box]**

Prefer not to say

I can't remember

Q19

Where did you/your household go to collect your puppy? [RANDOMISE LIST FOR EACH SURVEY TO AVOID BIAS]

1. The breeder's property – an outdoor kennels, barn or outbuilding
2. The breeder's property – from inside their home
3. The breeder's property – from outside their home, e.g. doorstep, garden
4. A lay-by
5. A car park
6. A service station
7. An airport
8. The breeder delivered my puppy to my property
9. Other (please specify) **[free text]**

Q20

[SURVEY LOGIC – If selecting option 1 or 2 above (Q19)]

What were your/your households first impressions of the environment your puppy was kept in? Please tell us below.

[free text]

Q21

Were you/your household comfortable with the place you received/collected your puppy from?

Yes

No (please explain) **[free text]**

Q22

Did you/your household see your puppy at a date previous to the day you brought them home?

Please select **all options** that apply

Yes – visited the breeder's property in person

Yes – saw my/our puppy on a live video call with their breeder

Yes – saw photos or a pre-recorded video of my/our puppy

No – did not ask to see my/our puppy

No – wanted to see my/our puppy but the breeder refused

No – other (please explain why) **[free text]**

Q23

[SURVEY LOGIC – If yes to option 1 or 2 above (Q22)]

How many times did you/your household see your puppy before you brought them home?

If you did not see them in person or via a live video call, then please enter '0' in that box.

Visits in person **[Number box]**

Live video calls **[Number box]**

Q24

On the day you brought your puppy home, which, if any other dogs did you see your puppy with?

Please select **all options** that apply

Their littermates

Other puppies (unsure if they were littermates)

Their mother

Their father

Another dog(s) they were not related to (e.g. another breed)

I only saw my/our puppy

I don't remember

I'm not sure, I wasn't the person who collected my/our puppy

Other (please specify) **[free text]**

Q25

How old were you told your puppy was when you brought them home?

Under 6 weeks old

7 to 8 weeks old

9 to 10 weeks old

11 to 12 weeks old

13 to 16 weeks old

I'm not sure/can't remember

Q26

Had your breeder provided your puppy with any of the following prior to you taking them home?

Please select **all options** that apply

Worming treatment

Flea treatment

Health check by a vet

Microchip

First vaccinations

Second vaccinations

Q27

Have you been in contact with your breeder since you brought your puppy/dog home?

Yes – I contacted them first

Yes – they contacted me first

No – I tried to contact them but got no response

No – I have not tried to contact them

Q28

Have you registered your puppy/dog with a vet?

1. Yes
2. No
3. No – not yet, but I intend to in the future

Q29

[SURVEY LOGIC – If yes to option 1 above (Q28)]

Have you taken your dog to a vet for any health problems since you brought them home?

No

Yes (please describe the health problem below) **[free text]**

Q30

Has your puppy/dog been vaccinated, or do you plan to in the future?

Yes – just their first vaccinations

Yes – first and second vaccinations

No – not yet, but I plan to in the future

No – not yet, I haven't decided

No – I have chosen not to vaccinate my puppy/dog and don't plan to in the future

Q31

Has your puppy/dog been neutered, or do you plan to have them neutered in the future?

Yes, aged under 6 months

Yes, aged over 6 months

No, but I intend to have them neutered when they are older

No, not yet, I haven't decided

No, but I do not plan to breed from them

No, because I plan to breed from them

Q32

Did you or someone in your household attend any puppy classes with your puppy/dog before they were 16 weeks old?

Yes, in-person puppy classes
Yes, online puppy classes
No, not as yet but I plan to before my puppy is 16 weeks old (if applicable)
No, I wanted to but there weren't any classes running
No, I do not intend to
No, other (please describe here) **[free text]**

Q33

Whilst your puppy was *under 16 weeks* old, did you deliberately leave them alone for any period of time to get them used to being left alone?

Yes
No
No, not as yet but I plan to before my puppy is 16 weeks old (if applicable)
I can't remember

Q34

In hindsight would you change anything about the way in which you acquired your puppy/dog (including your choice of breed and the source you purchased your puppy/dog from)?
Please describe in your own words
[free text]

Q35

Have you considered, or have you needed to, rehome your puppy/dog since you acquired them?

I still have my puppy/dog and have not considered rehoming them
I still have my puppy/dog, but I have considered, or I am currently considering rehoming them
I have rehomed my puppy/dog to another person/family
I have given my puppy/dog to a rehoming organisation/charity
N/A – My puppy/dog has passed away
N/A – My puppy/dog was put to sleep
Other (please specify) **[free text]**

Q36
<u>[SURVEY LOGIC – If yes to option 2 or 3 above (Q35)]</u>
<u>If you are comfortable doing so, please describe in your own words why you have rehomed, or thought about rehoming your puppy/dog</u>
[free text]

<u>FILTER:</u>

Q37
Yes, I would like to complete more questions, and I bought my puppy home **<u>FROM</u>** 23rd March 2020 onwards **[SURVEY LOGIC – send to Q38 and then to Q66]**
Yes, I would like to complete more questions, and I bought my puppy home **<u>BEFORE</u>** 23rd March 2020 **[SURVEY LOGIC – send to Q52 and then to Q70]**
No, I do not have time to complete more questions about my puppy's purchase, please take me to the end of the survey (I bought my puppy home **<u>FROM</u>** 23rd March 2020 onwards) **[SURVEY LOGIC – send to Q66]**
No, I do not have time to complete more questions about my puppy's purchase, please take me to the end of the survey (I bought my puppy home **<u>BEFORE</u>** 23rd March 2020) **[SURVEY LOGIC – send to Q70]**

<u>2020 EXTENDED QUESTIONS (POST 23/3/2020 OWNERS WITH TIME):</u>

Q38
<u>If you live in a multi-person household, who was the driving force in wanting to acquire a puppy?</u>
<u>Please select **all options** that apply</u>
Myself
Another adult in the household
A child or children in the household
All members of the household were equal in their desire to want a puppy
N/A
Other (please tell us who here) **[free text]**

Q39
<u>Did you feel pressured by your puppy's breeder to commit to buying your puppy?</u>
Yes
No
I'm not sure
I don't remember

Q40

Did you join a waiting list for your puppy?

Yes

No

I don't remember

Q41

After finding your puppy did you put down a deposit to secure him/her?

Yes – before I saw my puppy

Yes – after I saw my puppy

No – I was asked to but refused

No – I was not asked to

I don't remember

Other (please describe here) **[free text]**

Q42

Did your breeder have more than one litter of puppies for sale when you bought your puppy?

Yes – of the same breed/crossbreed

Yes – of more than one breed/crossbreed

No

I'm not sure

I don't remember

Q43

Did your breeder provide you with any of the following items when you collected your puppy?

Column

Yes

No

Not applicable

I'm not sure/can't remember

Row

Puppy's microchip details

Puppy's vaccinations record

Kennel Club change of ownership form

Copy of your puppy's pedigree (family tree)

Food they had been eating at the breeders

Feeding guidance in writing

The Puppy Contract

The Puppy's passport

Q44

Did you ask your breeder to see any information related to health testing of your puppy's parents?

N.B. health tests are not available for all dog breeds

Columns

Yes, and they provided me with it

Yes, but they couldn't provide it

No, I did not ask about this

No, I do not believe there are any tests available for my puppy's breed/crossbreed

Rows

The results of DNA (genetic) tests

The results of veterinary screening tests (e.g. hips, elbows, knees, eyes, respiratory testing)

Q45

Has your puppy's breeder offered you any of the following (at any point in time)?

Please select **all options** that apply

Advice on your puppy's health

Advice on your puppy's training/behaviour

Advice on your puppy's diet

Advice on your puppy's exercise regime

The option to return your puppy to them in the future for any reason

The option to board your puppy with them when on holiday

None of the above

Q46

Soon after you brought your puppy home, did you notice any of the following?

Please select **all options** that apply **[FLIP CHOICES FOR EACH SURVEY TO AVOID BIAS]**

Runny eye(s)

Runny faeces and/or diarrhoea

Being sick (vomiting)

Worms in faeces

Fleas/other parasites visible in fur/on skin

Hair loss

Wounds/sore areas of skin

Frequent itching/licking

Coughing

None of the above

Other (please specify) **[free text]**

Q47

Does your puppy currently have any health issues that you are concerned about?

No

Yes (please specify) **[free text]**

N/A – I no longer have my puppy

Q48

Did your puppy meet any *people from outside your household* between you buying them and reaching 16 weeks of age?

Yes

No

I'm not sure/can't remember

No, not as yet but I plan to before my puppy is 16 weeks old (if applicable)

Q49

Did your puppy meet any *dogs from outside your household* between you buying them and reaching 16 weeks of age?

Yes

No

I'm not sure/can't remember

No, not as yet but I plan to before my puppy is 16 weeks old (if applicable)

Q50

Did your puppy encounter any of the following experiences between you buying them and reaching 16 weeks of age?

Please select **all options** that apply

Column

Yes

No

I'm not sure/can't remember

No, not as yet but I plan to before my dog is 16 weeks old (if applicable)

Row

Walking in a public space (i.e. outside of your home/garden)

Visitors to their home

Fireworks

Thunderstorm

Walking near traffic

Travelling in a car

Dog groomer

Q51

Does your puppy currently show any of the following behaviours that you/your household find problematic?

Please select **all options** that apply **[FLIP CHOICES FOR EACH SURVEY TO AVOID BIAS]**

Pulling on their lead
Jumping up at people
Barking at other dogs
Not coming back when called
Fear of loud sounds (e.g. fireworks, thunderstorms)
Chasing, e.g. cats, wildlife
Clinginess (e.g. following you, sitting close)
Anxiety/fear around other dogs
Anxiety/fear around people in your household (including you)
Anxiety/fear around unfamiliar people
Aggression towards other dogs
Aggression towards people in your household (including you)
Aggression towards unfamiliar people
Guarding of food, toys, or other items
Mouthing
Barking or howling when left alone
Being destructive when left alone
Toileting (weeing or pooing) in the house when left alone
N/A – I no longer have my puppy
None of the above

2019 EXTENDED QUESTIONS (PRE 23/3/2020 OWNERS WITH TIME):

Q52

If you live in a multi-person household, who was the driving force in wanting to acquire a dog?

Please select **all options** that apply

Myself
Another adult in the household
A child or children in the household
All members of the household were equal in their desire to want a dog
N/A
Other (please tell us who here) **[free text]**

Q53

<u>Did you feel pressured by your dog's breeder to commit to buying your dog?</u>

Yes

No

I'm not sure

I don't remember

Q54

<u>Did you join a waiting list for your dog?</u>

Yes

No

I don't remember

Q55

<u>After finding your dog did you put down a deposit to secure him/her?</u>

Yes – before I saw my dog

Yes – after I saw my dog

No – I was asked but refused

No – I was not asked to

I don't remember

Other (please describe here **[free text]**

Q56

<u>Did your breeder have more than one litter of puppies for sale when you bought your dog?</u>

Yes – of the <u>same</u> breed/crossbreed

Yes – of <u>more than one</u> breed/crossbreed

No

I'm not sure

I don't remember

Q57

Did your breeder provide you with any of the following items when you collected your dog?

Column

Yes

No

Not applicable

I'm not sure/can't remember

Row

Dog's microchip details

Dog's vaccinations record

Kennel Club change of ownership form

Copy of your dog's pedigree (family tree)

Food they had been eating at the breeders

Feeding guidance in writing

The Puppy Contract

Dog's passport

Q58

Did you ask your breeder to see any information related to health testing of your dog's parents?

N.B. health tests are not available for all dog breeds

Columns

Yes, and they provided me with it

Yes, but they couldn't provide it

No, I did not ask about this

No, I do not believe there are any tests available for my dog's breed/crossbreed

Rows

The results of DNA (genetic) tests

The results of veterinary screening tests (e.g. hips, elbows, knees, eyes, respiratory testing)

Q59

Has your dog's breeder offered you any of the following (at any point in time)?

Please select **all options** that apply

Advice on your dog's health

Advice on your dog's training/behaviour

Advice on your dog's diet

Advice on your dog's exercise regime

The option to return your dog to them in the future for any reason

The option to board your dog with them when on holiday

None of the above

Q60

Soon after you brought your dog home, did you notice any of the following?

Please select **all options** that apply **[FLIP CHOICES FOR EACH SURVEY TO AVOID BIAS]**

Runny eye(s)
Runny faeces and/or diarrhoea
Being sick (vomiting)
Worms in faeces
Fleas/other parasites visible in fur/on skin
Hair loss
Wounds/sore areas of skin
Frequent itching/licking
Coughing
None of the above
Other (please specify) **[free text]**

Q61

Does your dog currently have any health issues that you are concerned about?

No
Yes (please specify) **[free text]**
N/A – I no longer have my dog

Q62

Did your dog meet any *people from outside your household* between you buying them and reaching 16 weeks of age?

Yes
No
I'm not sure/can't remember

Q63

Did your dog meet any *dogs from outside your household* between you buying them and reaching 16 weeks of age?

Yes
No
I'm not sure/can't remember

Q64

Did your dog encounter any of the following experiences between you buying them and reaching 16 weeks of age?

Please select **all options** that apply

Column

Yes

No

I’m not sure/can’t remember

Row

Walking in a public space (i.e. outside of your home/garden)

Visitors to their home

Fireworks

Thunderstorm

Walking near traffic

Travelling in a car

Dog groomer

Q65

Does your dog currently show any of the following behaviours that you/your household find problematic?
Please select **all options** that apply **[FLIP CHOICES FOR EACH SURVEY TO AVOID BIAS]**

Pulling on their lead
Jumping up at people
Barking at other dogs
Not coming back when called
Fear of loud sounds (e.g. fireworks, thunderstorms)
Chasing, e.g. cats, wildlife
Clinginess (e.g. following you, sitting close)
Anxiety/fear around other dogs
Anxiety/fear around people in your household (including you)
Anxiety/fear around unfamiliar people
Aggression towards other dogs
Aggression towards people in your household (including you)
Aggression towards unfamiliar people
Guarding of food, toys, or other items
Mouthing
Barking or howling when left alone
Being destructive when left alone
Toileting (weeing or pooing) in the house when left alone
N/A – I no longer have my puppy
None of the above

2020 COVID SPECIFIC QUESTIONS (POST 23/3/2020 OWNERS WITH AND WITHOUT TIME):

Q66

Had you or someone in your household considered buying a puppy before the COVID-19 pandemic?
Yes
No
I'm not sure

Q67

Do you feel that the COVID-19 pandemic influenced your decision to purchase a puppy?
Yes
No
I'm not sure

Q68
[SURVEY LOGIC – If yes to option 1 above (Q67)]
What were the reasons that the COVID-19 pandemic influenced you/your households' decision to purchase a puppy?
Please select **all options** that apply **[RANDOMISE LIST FOR EACH SURVEY TO AVOID BIAS]**
I/we wanted more company due to being at home more
I/we had more time to care for a dog
I/we wanted more company as family and/or friends were unable to visit me/us
I/we wanted a reason to go outside to exercise more
I/we wanted something happy to focus on
I/we were bored due to the restrictions imposed by lockdown
I/we had extra money to spend that I/we would have usually spent on other things
My child/children were at home and I/we wanted something to keep them busy
Other (please tell us why here) **[free text]**

Q69
When you decided to buy a puppy during the COVID-19 pandemic, did you foresee any additional challenges to owning your puppy in the future (e.g. after restrictions such as lockdown ended)?
Please tell us in your own words below
[free text]

CORE DEMOGRAPHIC QUESTIONS (FOR ALL OWNERS):

Q70
Are you the primary carer for your puppy/dog (i.e. the person in your household that provides your puppy/dog with the majority of care such as feeding and walking)?
Yes
No
I share the role of primary carer for my puppy/dog with someone else in the household
I share the role of primary carer for my puppy/dog with someone else in a different household
N/A – I no longer have my puppy/dog

Q71

How old are you?

18 to 24 years old
25 to 34 years old
35 to 44 years old
45 to 54 years old
55 to 64 years old
65 to 74 years old
75 years old or older

Q72

What is your gender?

Female
Male
Other
Prefer not to say

Q73

If you are happy to, please provide the first three/four digits of your postcode (e.g. AL9)

[free text]

Q74

Were you/your household affected by the COVID-19 pandemic in any of the following ways?

Please select **all options** that apply

One or more members of my household/I was furloughed
One or more members of my household/I started working from home
One or more members of my household/I became unemployed
Child(ren) had to be homeschooled or cared for from home
None of the above
Prefer not to say

Q75

Were you or any member of your household classed as a key worker during the COVID-19 lockdown?

Please select **all options** that apply

Yes, I am a key worker
Yes, another member of my household is a key worker
No
I prefer not to say
I'm not sure

Q76

Are you or any member of your household employed in the canine and/or animal care sector (e.g. veterinary nurse, dog groomer, dog trainer, etc.)?

Yes

No

I'm not sure

Q77

[SURVEY LOGIC – If yes to option 1 above (Q76)]

Which canine and/or animal care sector are you or a member of your household employed in?

Please select **all options** that apply **[RANDOMISE LIST FOR EACH SURVEY TO AVOID BIAS]**

Veterinary surgeon

Veterinary nurse

Animal care assistant

Veterinary scientist

Dog behaviourist

Dog trainer

Dog daycare/boarding kennels

Dog walker

Dog groomer

Rehoming center staff

Other (please specify) **[free text]**

Q78

Did you grow up with a dog in your childhood home?

Yes

No

Q79

As an adult, have you ever owned or co-owned a dog before you purchased your puppy/dog?

Yes

No – but someone else in my household has

No – I am/everyone in my household is a first-time dog owner(s)

Q80

What best describes your current living situation?

Live alone

Live in an adult only home (over 18 years old)

Live in a home with adults and children

Live in a home with children where I am the only adult

Other (please specify) **[free text]**

Q81

[SURVEY LOGIC – If yes to option 3 or 4 above (Q80)]

How old are the children that your dog/puppy currently shares a home with?

Please select **all options** that apply

Under 5 years old

5-10 years old

11-15 years old

16-18 years old

Q82

How many (if any) other dogs currently live in the same home as your puppy/dog?

Please enter a whole number

N.B. If they are the only dog in your household, please type '0'

[Number box]

Q83

Does your puppy/dog currently have access to outdoor space?

Yes – a garden or yard (private)

Yes – a garden or yard (shared)

No

N/A – I no longer have my puppy/dog

Q84

Is your puppy/dog *currently* left at home alone for more than four hours without being taken out for exercise or having someone come in to check on them?

Yes

No

I'm not sure

N/A – I no longer have my puppy/dog

Q85

<u>In *the future*, is your puppy/dog likely to be left at home alone for more than four hours without being taken out for exercise or having someone come in to check on them?</u>

Yes

No

I'm not sure

N/A – I no longer have my puppy/dog

Q86

<u>How much do you expect your puppy/dog to cost per year? Please consider all ongoing costs, for example food, equipment and veterinary bills for preventative healthcare such as worming (but NOT illness/injury).</u>

£ (please state as a single number, not a range) **[Number box]**

I'm not sure

N/A – I no longer have my puppy/dog

Q87

<u>We may wish to get in contact with you in the future for limited reasons outlined below.</u>

<u>Please let us know which (if any) you are happy to be contacted about, and provide us with your preferred email address:</u>

To be asked to clarify my responses to this study

To be sent the results of this study

To be invited to take part in further research about my puppy/dog

None of the above

Comment box: **[email address]**

<u>DEBRIEF QUESTIONS (ALL OWNERS):</u>

Q88

<u>Had you heard of the Petfished Campaign before purchasing your puppy?</u>

Yes

No

Q89

<u>Had you heard of The Puppy Contract before purchasing your puppy?</u>

Yes

No

Q90

[SURVEY LOGIC – If yes to option 1 above (Q89)]

Did you use The Puppy Contract when purchasing your puppy?

If you did not on this occasion, please explain in your own words why you chose not to or were unable to

Yes

No (please describe why) **[free text]**
