# Supplementary material for: Pandemic Puppies: Demographic Characteristics, Health and Early Life Experiences of Puppies Acquired during the 2020 Phase of the COVID-19 Pandemic in the UK
Source: Animals (Basel). 2022 Mar 2;12(5):629. doi: 10.3390/ani12050629 (PMC8909199; doi:10.3390/ani12050629)
Supplement: Supplementary file 1 [file animals-12-00629-s001.zip › animals-1606453-supplementary/Brand et al File S2 File Qualitative Coding Framework ¿C Puppy Classes.pdf]

Qualitative Coding Framework for Content Analysis

The entire data set consisting of 6527 valid responses was subjected to content analysis at the same time, i.e., additionally including those puppies purchased between 1 January and 22 March for both years.
In the case of this question (Q32), where only one response was requested, the first comment in the free-text was either assigned to a new category or back allocated to an existing category.

Back Allocating to Existing Deductive Category:
Free-text responses were allocated to the existing deductive categories (MCQ choices) if not selected by the respondent.

Miscellaneous free-text response:
Free-text responses which did not explicitly answer the question were classified as 'Miscellaneous' and were not included in the analysis.

Uninterpretable:
Free-text responses consisting of random digits or punctuation that didn't appear to mean anything were classified as 'uninterpretable' and not included in the analysis.

Creating New Inductive Categories:
Where comments did not correlate to the existing deductive categories (MCQ choices), new categories were created as described by Packer *et al*., Pandemic Puppies: Characterising motivations and behaviours of UK owners who purchased puppies during the 2020 COVID-19 Pandemic. *Animals*. **2021**, DOI 10.3390/ani11092500 (section 2.5. Qualitative Content Analysis of Free-Text Options).

The following tables give details of both the existing and new categories for the question analysed for this publication along with examples of comments.

Q32: Did you or someone in your household attend any puppy classes with your puppy/dog before they were 16 weeks old?

- No, other (please describe here) (n=696)

Back Allocating to Existing Deductive Category	Example(s) Back Allocated
Yes, in-person puppy classes	*"1:1 trainer at home", "One to one puppy training - trainer came to our house", "Trainer came to our house as the classes were all full"*
Yes, online puppy classes	*"I am in an online training group", "I'm currently doing one online", "My dog trainer that I had used previously had put her classes on line which was a great refresher for me"*
No, not as yet but I plan to before my puppy is 16 weeks old (if applicable)	*"I will if I can my puppy is 9 weeks old", "She is only 14 weeks old and we intend to enroll in an in-person puppy class.", "She is only 11wks old. Intend to, pandemic dependent"*
No, I wanted to but there weren't any classes running	*"Cancelled after 2 weeks due to COVID", "Booked classes but had to cancel due to local lockdown", "The waiting lists were so long because everyone had bought puppies that I couldn't get onto one.", "There is a lack of puppy classes due to lockdown"*
No, I do not intend to[1]	*"He was regularly interacting with other dogs. Without going to one of the overbearing ridiculous puppy classes.", "Don't feel necessary to go to puppy classes, confident in training and have friends and family that have dogs that will be used for socialisation. Unfortunately, puppy classes aren't cheap", "Been to so many in the past", "I watched training videos online and implemented myself", "I use a dog training group on Facebook which is specific to boxers, and it's really good"*

[1] Responses mentioning that puppy classes are unnecessary, etc., were back allocated here, along with responses that mentioned that they were an experienced dog owner. In addition, responses that mentioned using Facebook groups, online videos/YouTube, and implementing what they learnt themselves, were back allocated to this category.

New Category	Example(s)
No, I chose not to because I am a dog professional[2]	*"I am a dog trainer myself and she got lots of opportunities to socialise with clients dogs etc", "I am a canine professional and do her training myself."*
No, I was unable to attend before 16 weeks due to my puppy's circumstances (e.g., age at purchase close to 16 weeks, timing of vaccinations, poor health <16 weeks)	*"Due to puppy strangles and couldn't get 2nd vaccine due to being on antibiotics and steroids for over a month and the pandemic he was 5 months before he got his 2nd vaccine as he had to get his 1st one again. So didn't interact with any dogs until he was over 5 months.", "Distance to classes made car travel necessary and puppy suffered from car sickness. I decided the stress of travel would outdo any benefit from the classes."*
No, I was unable to attend classes while my puppy was <16 weeks due to my own circumstances (e.g., health, work commitments)	*"I'm working full time and the class times near me were not suitable.", "Classes at the vet practice were unfortunately too late in the evening (after 7).", "I work in a school and want to limit my contacts during the COVID pandemic…", "I was not well at the time"*
No, my puppy died <16 weeks	*"Died", "No our puppy died within a week of bringing it home of Parvo", "She didn't make it to 9 weeks old."*
Miscellaneous	*"Haven't decided whether to or Not"*

[2] Although these could have been back allocated to 'No, I do not intend to' it was felt that experience of dog training in a professional capacity warranted its own category.
