# Supplementary material for: Pandemic Puppies: Demographic Characteristics, Health and Early Life Experiences of Puppies Acquired during the 2020 Phase of the COVID-19 Pandemic in the UK
Source: Animals (Basel). 2022 Mar 2;12(5):629. doi: 10.3390/ani12050629 (PMC8909199; doi:10.3390/ani12050629)
Supplement: Supplementary file 1 [file animals-12-00629-s001.zip › animals-1606453-supplementary/Brand et al File S3 File Coding Framework VetCompassTM Disorder terms.pdf]

Qualitative Coding Framework for assigning the free-text responses to VetCompass™ disorder terms

The entire data set consisting of 6527 valid responses was assigned to VetCompass™ disorder terms at the same time, i.e., additionally including those puppies purchased between 1 January and 22 March for both years.
In the case of this question (Q46/Q60), where multiple responses were allowed, all relevant comments were assigned to VetCompass™ disorder terms

The following tables give details of the VetCompass™ disorder terms for the question assigned for this publication along with examples of comments.

Q46/Q60: Soon after you brought your puppy home, did you notice any of the following?

- Other (please specify) (n=219)

**VetCompass™ Disorder Term**	**Example(s)**
Abdominal disorder	*"Swollen tummy"*
Anal sac disorder	*"Compacted anal glands…", "Anal glands needed expression"*
Appetite disorder	*"Lack of appetite", "She hardly ate for days…"*
Collapsed	*"There was an issue, there was concern that the litter overheated on the way to vets for first vaccination & chipping"*
Complication associated with clinical care procedure	*"This was to do with previous operation…"*
Drug therapy adverse reaction	*"He was on anti biotic when he came to us but then required more"*
Ear (aural) disorder	*"Ear infection", "After a week the puppy had a red ear…"*
Enteropathy	*"Parvovirus", "Giardia", "Blood in stools", "Being sick…"*
Female reproductive abnormality	*"Vaginal discharge", "Vaginitis"*
Foreign body	*"Foreign body was suspected"*
Haematopoietic system disorder	*"Blood from rectum"*
Hearing impaired/deafness	*"The pup is deaf"*
Heart (cardiac) disease	*"A loud heart murmur…", "Heart condition"*
Hernia	*"umbilical hernia", "He needed a hernia operation…"*
Lethargy	*"Extreme tiredness", "Lack of energy"*
Lower respiratory tract disorder	*"…chest…",*
Mass/lump/swelling	*"…swollen eye…"*
Musculoskeletal disorder	*"Diagnosed with loose hip joint…", "Lameness…"*
Ophthalmological disorder	*"Conjunctivitis", "Entropion", "Inflamed eyelids"*
Oral cavity (mouth) disorder	*"Hair lip",*
Parasite infestation	*"Ear mites", "She had walking dandruff."*
Polyuria/Polydipsia	*"Constant drinking", "Excessive weeing"*
Skin (cutaneous) disorder	*"Itchy", "Dandruff", "Flaky skin", "…foliculitus…"*
Tail disorder	*"… kinked tail"*
Thin/underweight	*"Underweight", "Was quite thin"*
Traumatic injury	*"Broken tail", "…he had a broken leg…"*
Undesirable behaviour disorder	*"…we believe this is anxiety related", "…anxiety of being left alone for short periods."*
Upper respiratory tract disorder	*"…coughing"*
Urinary system disorder	*"Frequent urine infections", "Urinary track infection"*
Vascular disorder	*"…abherrent left subclavian aortic arch"*
